# Azathioprine versus Beta Interferons for Relapsing-Remitting Multiple Sclerosis: A Multicentre Randomized Non-Inferiority Trial

**DOI:** 10.1371/journal.pone.0113371

**Published:** 2014-11-17

**Authors:** Luca Massacesi, Irene Tramacere, Salvatore Amoroso, Mario A. Battaglia, Maria Donata Benedetti, Graziella Filippini, Loredana La Mantia, Anna Repice, Alessandra Solari, Gioacchino Tedeschi, Clara Milanese

**Affiliations:** 1 Dipartimento di Neuroscienze, Psicologia, Farmaco e Salute del Bambino Università di Firenze, Firenze, Italy; 2 Neurologia 2, Azienda Ospedaliero-Universitaria Careggi, Firenze, Italy; 3 Fondazione IRCCS Istituto Neurologico Carlo Besta, Milano, Italy; 4 Dipartimento di Neuroscienze, Sezione di Farmacologia, Università Politecnica delle Marche, Ancona, Italy; 5 Associazione Italiana Sclerosi Multipla (AISM), Fondazione Italiana Sclerosi Multipla (FISM), Genova, Italy; 6 Dipartimento Universitario di Neurologia, Azienda Ospedaliera Universitaria Integrata di Verona, Verona, Italy; 7 Unità di Neurologia - Multiple Sclerosis Center, I.R.C.C.S. Santa Maria Nascente Fondazione Don Gnocchi, Milano, Italy; 8 Clinica Neurologica, Università di Napoli, Napoli, Italy; Charite - Universitätsmedizin Berlin, Germany

## Abstract

For almost three decades in many countries azathioprine has been used to treat relapsing-remitting multiple sclerosis. However its efficacy was usually considered marginal and following approval of β interferons for this indication it was no longer recommended as first line treatment, even if presently no conclusive direct β interferon-azathioprine comparison exists. To compare azathioprine efficacy versus the currently available β interferons in relapsing-remitting multiple sclerosis, a multicenter, randomized, controlled, single-blinded, non-inferiority trial was conducted in 30 Italian multiple sclerosis centers. Eligible patients (relapsing-remitting course; ≥2 relapses in the last 2 years) were randomly assigned to azathioprine or β interferons. The primary outcome was annualized relapse rate ratio (RR) over 2 years. Key secondary outcome was number of new brain MRI lesions. Patients (n = 150) were randomized in 2 groups (77 azathioprine, 73 β interferons). At 2 years, clinical evaluation was completed in 127 patients (62 azathioprine, 65 β interferons). Annualized relapse rate was 0.26 (95% Confidence Interval, CI, 0.19–0.37) in the azathioprine and 0.39 (95% CI 0.30–0.51) in the interferon group. Non-inferiority analysis showed that azathioprine was at least as effective as β interferons (relapse RR_AZA/IFN_ 0.67, one-sided 95% CI 0.96; p<0.01). MRI outcomes were analyzed in 97 patients (50 azathioprine and 47 β interferons). Annualized new T2 lesion rate was 0.76 (95% CI 0.61–0.95) in the azathioprine and 0.69 (95% CI 0.54–0.88) in the interferon group. Treatment discontinuations due to adverse events were higher (20.3% vs. 7.8%, p = 0.03) in the azathioprine than in the interferon group, and concentrated within the first months of treatment, whereas in the interferon group discontinuations occurred mainly during the second year.

The results of this study indicate that efficacy of azathioprine is not inferior to that of β interferons for patients with relapsing-remitting multiple sclerosis. Considering also the convenience of the oral administration, and the low cost for health service providers, azathioprine may represent an alternative to interferon treatment, while the different side effect profiles of both medications have to be taken into account.

**Trial Registration:**

EudraCT 2006-004937-13

## Introduction

For almost three decades azathioprine (AZA) has been used in many countries to treat relapsing-remitting multiple sclerosis (MS) based on placebo controlled randomized clinical trials (RCTs) [1]–[4]. Efficacy however was usually considered marginal [5], [6], and following approval of β interferons (IFNs) AZA was no longer recommended as first-line therapy [7]. Lack of MRI evaluation, methodological weaknesses and the low power of the trials may have fostered perception of the poor efficacy of AZA, whereas consistently efficacious and safe IFN trials in MS [8]–[11] have made IFN a drug of choice for this indication [7]. However, meta-analyses [12]–[14], new comparative RCTs [15], [16], and MRI results [17], [18] suggest a similar effect size of AZA in relapsing-remitting MS. Presently no conclusive direct IFN-AZA comparison exists. This paper documents an independent multicenter RCT evaluating the non-inferiority of the efficacy of AZA vs. IFNs on clinical and MRI measures of disease activity in relapsing-remitting MS.

## Materials and Methods

The protocol for this trial and supporting CONSORT checklist are available as supporting information; see Protocol S1, Amendment S1, and Checklist S1.

### Ethics statement

This study was approved by ethics committees in the coordinating center (Careggi University Hospital, Ethic Committee, Florence) and in each of the participating centers (**Fondazione IRCCS Istituto Neurologico Carlo Besta**, Milano; **Clinica Neurologica**, Novara; **Università “La Sapienza”**, Roma; **Policlinico “G. Rodolico” Azienda Ospedaliero-Universitaria**, Catania; **Clinica Neurologica 2**, Genova; **Azienda Ospedaliera Universitaria Integrata**, Verona; **Ospedale Clinicizzato “Colle Dall'Ara”**, Chieti; **Università di Sassari**, Sassari; **Università di Napoli**, Napoli; **Ospedale S. Antonio**, Padova; **Ospedale Civile S. Agostino-Estense**, Modena; **Ospedale Santa Maria**, Reggio Emilia; **Policlinico Universitario Mater Domini**, Catanzaro; **Ospedale S. Gerardo**, Monza; **Azienda Ospedaliero-Universitaria S. Anna**, Ferrara; **Ospedali Riuniti**, Ancona; **Istituto S. Raffaele “G. Giglio”**, Cefalù; **Azienda Ospedaliero San Giovanni Battista**, **Università di Torino**, Torino; **Ospedale Sacro Cuore**, Negrar; **Ospedale Santa Chiara**, Trento; **Ospedale Regionale**, Bolzano; **Azienda Ospedaliero-Universitaria Senese**, **Policlinico “Le Scotte”**, Siena; **Ospedale “Misericordia e Dolce”**, Prato; **Università degli Studi di Pisa**, Pisa; **Policlinico “G. Martino”**, Messina; **Università degli Studi di Palermo**, Palermo; **Università Cattolica**, **Policlinico Gemelli**, Roma; **Dipartimento Neuroriabilitativo ASL CN1**, Cuneo; **Luigi Gonzaga Hospital**, Orbassano Ethics Committees), adhered to Good Clinical Practice (GCP) guidelines and Declaration of Helsinki. The original trial was registered in 2006 in the EudraCT register (EUDRACT n.: 2006-004937-13) at a time that was prior to being accepted as a registry that fulfills the requirements by the International Committee of Medical Journal Editors (ICMJE) (http://www.icmje.org/faq_clinical.html). Since this registry was only considered to fulfill the requirements by the ICMJE since June 2011 and was not publicly available for several years after it was established, this precluded fulfilment of the requirements outlined by the ICMJE. We confirm that all ongoing and future trials are now registered.

### Study design and patients

Designed as a multicenter, randomized, single-blinded, phase III clinical trial, the study assesses non-inferiority of AZA efficacy vs. IFNs over two years. Patients were recruited between February 2007 and March 2009 in 30 MS centers throughout Italy. Inclusion criteria were: age, 18–55 years; relapsing-remitting MS [19]; at least two clinical relapses in the preceding two years; a baseline Expanded Disability Status Scale (EDSS) [20] score from 1.0 to 5.5; effective female contraception and a signed informed consent. Exclusion criteria were: clinical relapses or steroid therapy 30 days prior to study entry; immunomodulatory or immunosuppressive treatments in the preceding year; concomitant diseases precluding IFN or AZA treatment; pregnancy or breastfeeding; cognitive decline preventing informed consent; pathological conditions interfering with MS evolution; non-steroidal anti-inflammatory drugs (NSAID) allergy or intolerance to AZA or IFNs.

The study was an independent academic initiative supported by the Italian Medicine Agency (Agenzia Italiana del Farmaco, AIFA) through a competitive Grant following a public call aimed to support independent Clinical Trials.

### Randomization and blinding

Patients were selected for AZA or IFNs using a computer generated central randomization list (1∶1 ratio), in blocks of four and stratified by disability score (EDSS≤3.5 or >3.5). Patients were assessed by an unblinded treating and a blinded examining neurologist at their centers. Brain MRI images were centrally analyzed by two blinded independent experts at the Image Analysis Centre of the University of Florence (Italy).

### Interventions

Treatment was prescribed free of charge by treating neurologists and self-administered within one month after screening and one week after randomization.

#### Standard treatment

The IFN-treated patients were either administered 250 µg of IFNβ-1b subcutaneously on alternate days (Betaferon), 30 µg of IFNβ-1a IM, weekly (Avonex); 22/44 µg of subcutaneous IFNβ-1a thrice weekly (Rebif). The type of IFNβ (Betaferon, Avonex or Rebif) was selected by the treating neurologist. The standard dose was titrated over the first four weeks.

#### Experimental treatment

The AZA-treated patients were given an oral target dose of 3 mg/kg/day, individually adjusted to their differential white cell counts. The initial 50 mg/day dose was subsequently titrated for the first six to eight weeks, increasing 50 mg every fortnight to the target dose.

#### Treatment adjustment and discontinuation criteria

For all medications, treatment adjustment criteria included: reaching grade two for adverse events (AEs) of Common Toxicity Criteria (CTC) [21], including n<800/µl lymphocyte count and n<3000/µl white blood cells. For AZA in case of grade two AEs, a 25/50 mg dose reduction was required. When the AE occurred during dose titration the higher dose was not prescribed. Returning to the target dose after reduction or increasing dose during titration was allowed for AEs occurring only once, otherwise the low dose was maintained. The treatment monitoring, including hemato-chemical tests (erythrocytes, hemoglobin, leukocytes with differential count, platelets, ALT, AST, GGT, ALP, and bilirubin), were performed quarterly. These tests were performed every fortnight during the first two months of treatment (one month for the IFNs) and when a grade two AE occurred. Treatment was discontinued for grade two AEs persistent at two subsequent controls after dose reduction. Other withdrawal criteria were: a grade three AE or AEs considered intolerable by patients or treating neurologists; treatment failure (i.e., more relapses during the study than in the previous two years, or an equal number of relapses and increase of at least one EDSS point confirmed after six months, or shift to a secondary progressive course); pregnancy; and consent withdrawal.

#### Co-interventions

Symptomatic treatments were allowed and 1 g of I.V. methylprednisolone was given for three-five days for relapses, as prescribed by the treating neurologist.

### Procedures

The treating neurologist oversaw the overall medical management of patients, including drug prescription and self-administration instruction, scheduled (quarterly) and unscheduled (i.e., at the onset of new symptoms or complications) follow-up visits where he/she recorded symptoms, blood test results, clinical AEs and their management, and any treatment decision, including discontinuation. The examining neurologist was responsible for the neurological examination and EDSS scoring at scheduled (every six months) and unscheduled visits, that were requested by the treating neurologist to confirm relapses. These included the onset of new neurological symptom(s), or worsening of pre-existing ones from MS, determining worsening of at least one point in one or more functional system or at least 0.5 EDSS points. A new symptom was considered part of a new relapse if it lasted at least 48 hours with no fever, and if reported at least 30 days from the end of a previous relapse. To discontinue treatment a final visit was planned within 30 days from the last dose.

A Contract Research Organization (CRO) visited all centers before enrolment and every four months thereafter.

### Outcomes

#### Clinical efficacy

The primary outcome was annualized relapse rate ratio (RR) over two years. Secondary clinical outcomes were: a) annualized relapse rate during the first and second year; b) proportion of patients with 0, 1, and ≥2 relapses during the first and second year; c) proportion of patients with corticosteroid-treated relapses; d) time to first relapse after randomization; e) proportion of patients with no confirmed disability progression, i.e., without an increase of at least one EDSS point confirmed after at least six months over two years; f) mean EDSS change from baseline to the end of follow-up; g) number of treatment failures; h) mean change of the MSQOL-54 scale [22] over two years.

#### Brain MRI

Brain lesions were evaluated through MRI scans performed over 30 days prior to treatment (baseline) and at two years (study completion). In the MRI study participated 23 Centers, all identified prior to the beginning of the study. The primary MRI outcome was the number of new T2 brain lesions, defined as new or enlarging lesions on T2-weighted scans. Secondary outcomes were: a) proportion of patients with 0, 1–2, ≥3 new T2 brain lesions; b) combined new and enhancing lesions (CE); c) mean and median Gadolinium contrast enhancing (Gd+) lesions on T1-weighted scans; d) proportion of patients with 0, 1–2, ≥3 Gd+ lesions. New lesion numbers were evaluated through dedicated software packages (Analyze 10.0), comparing each scan obtained at study completion with the corresponding baseline scan [see Methods S1 in File S1 for details].

#### Safety

Data was collected on: 1) AEs and serious AEs (SAEs); 2) patients with any AE; 3) patient withdrawal after any AE; 4) severity of any AE and their correlation with treatments as judged by the treating neurologist. Frequency and severity of AEs were actively assessed every three months or upon patient request. Severity was graded using the National Cancer Institute Common Terminology Criteria for AE [21]. SAE notification was sent to a specifically appointed Pharmacological Surveillance Unit (PSU).

### Non-inferiority margin, power and sample size

#### Non-inferiority margin

To compare treatment relapse rates, a non-inferiority margin (M) was calculated following published guidelines [23]–[25], as a fraction of the mean effect of IFNs vs. placebo (E_IFNvsPlacebo_) on the same outcome measure in previous trials with the same inclusion criteria and follow-up period [8], [9], [11]. By next expressing the E_IFNvsPlacebo_ as a relapse rate ratio, M was expressed as 50% of the excess to 1.0 of this rate ratio. Given the historical E_IFNvsPlacebo_ of 1.46 ( = 2.55/1.75, corresponding to the relapse rate reduction through IFN treatment), M = 1.23 was therefore selected [8], [9]. The annualized new T2 lesion rate over two years was chosen as the primary MRI outcome, as this was the main MRI outcome available in the pivotal trial aimed at establishing the efficacy of IFNβ-1b vs. placebo and whose inclusion criteria and follow-up length were identical [8], [11], thereby enabling precise evaluation of the E_IFNvsPlacebo_ on new T2 lesion rates, as their ratio was 2.67 ( = 6.4/2.4). Based on these data, a non-inferiority margin of M = 1.84 was established *a priori*, as 50% of the excess to 1.0 of the 2.67 historical ratio.

#### Power and sample size

Sample size was calculated to verify the non-inferiority of AZA against IFNs. With a power of 80%, α of 5% and under the hypothesis of no difference between the means of relapse rates (new T2 lesion rates for MRI), with an expected loss of 20% at follow-up, 360 patients (175/treatment arm) for relapse, and 192 patients (96/treatment arm) for MRI were needed. However, the sample size of the study was undermined by the revision of the Italian National Health System reimbursement criteria, that occurred during the recruitment period and allowed IFN therapy from the first MS attack, thus overcoming the required presence of at least two relapses during the previous two years, which was one of the inclusion criteria of this study. This change remarkably reduced the number of eligible patients and the recruitment slowed to such a low rate that the Steering Committee of the study judged the planned sample size not feasible any more. For this reason a protocol amendment, approved by the Independent Data and Safety Management Committee (IDSMC) and by the Ethic Committee of the Coordinating Center, recommended a 150 patient recruitment ceiling, accepting a power of 60–65% for relapses, and 80% for MRI outcome, under the hypothesis of no differences between the means of relapse/new T2 lesion rates [see Protocol S1 and Amendment S1 for details]. It is worth to note that the request of amendment was submitted by the Steering Committee exclusively on the basis of the observed accrual rate, when no data or codes were available.

### Statistical analyses

#### Baseline characteristics

Baseline clinical and demographic characteristics were analyzed using χ^2^ test for categorical, and t-test (or Mann-Whitney test in the absence of Normal distribution) for continuous variables.

#### Clinical outcome measures

AZA efficacy was judged non-inferior to IFNs if the upper limit (U_L_) of the one-sided 95% confidence interval (95% CI) of the annualized relapse RR_AZA/IFN_ over two years, calculated by Poisson regression, was <M = 1.23. Secondary outcomes were analyzed using χ^2^ test with one degree of freedom for rate comparison (based on Poisson regression); χ^2^ test with two degrees of freedom for number of relapsed patients; Kaplan-Meier curves, log-rank test and Cox proportional-hazards model for time to first relapse; Fisher's exact test for patients with no confirmed disability progression; and t-test for EDSS and MSQOL-54 score changes. For the annualized relapse rate, sensitivity analyses were performed adjusting for baseline covariates (number of relapses during the previous two years, baseline EDSS score, and disease duration from onset of symptoms), and excluding Avonex treated patients. An additional sensitivity analysis was performed to include in the analysis patients lost to follow-up, using two multiple imputation methods (monotone logistic regression and fully conditional specification [FCS] logistic regression method) [26]–[28], taking the randomized treatment as the covariate (i.e., incorporating possible different uncertainty due to different dropout rates between the two randomized treatment groups). All analyses were performed in the intention to treat (ITT) and per-protocol (PP, i.e. after excluding noncompliant patients and drop-outs) populations. In the analyses based on relapse rates and on proportion of patients with relapses or disability progression, patients lost to follow-up were excluded.

#### Brain lesions

AZAs were judged non-inferior to IFNs if the U_L_ of the one-sided 95% CI of the annualized new T2 lesion rate ratio over two years, calculated by Poisson regression, was <M = 1.84. Secondary outcomes were analyzed through χ^2^ test with one degree of freedom for rate comparison (based on Poisson regression); χ^2^ test with two degrees of freedom for number of patients with lesions; and Mann-Whitney test for Gd+ lesion number. All analyses were performed in the ITT and PP populations.

#### Adverse Events

AEs were analyzed as rates, in terms of patients with AEs and overall number of AEs, using χ^2^ test based on Poisson regression for rate comparison, and χ^2^ test for categorical variable comparison for discontinued interventions after AEs, AE severity and correlation of AE with treatment. SAEs were described reporting their postulated correlation with treatment and any consequent discontinuation.

Data were reported following the CONSORT guidelines [29].

## Results

### Characteristics of participants

**Figure 1:**
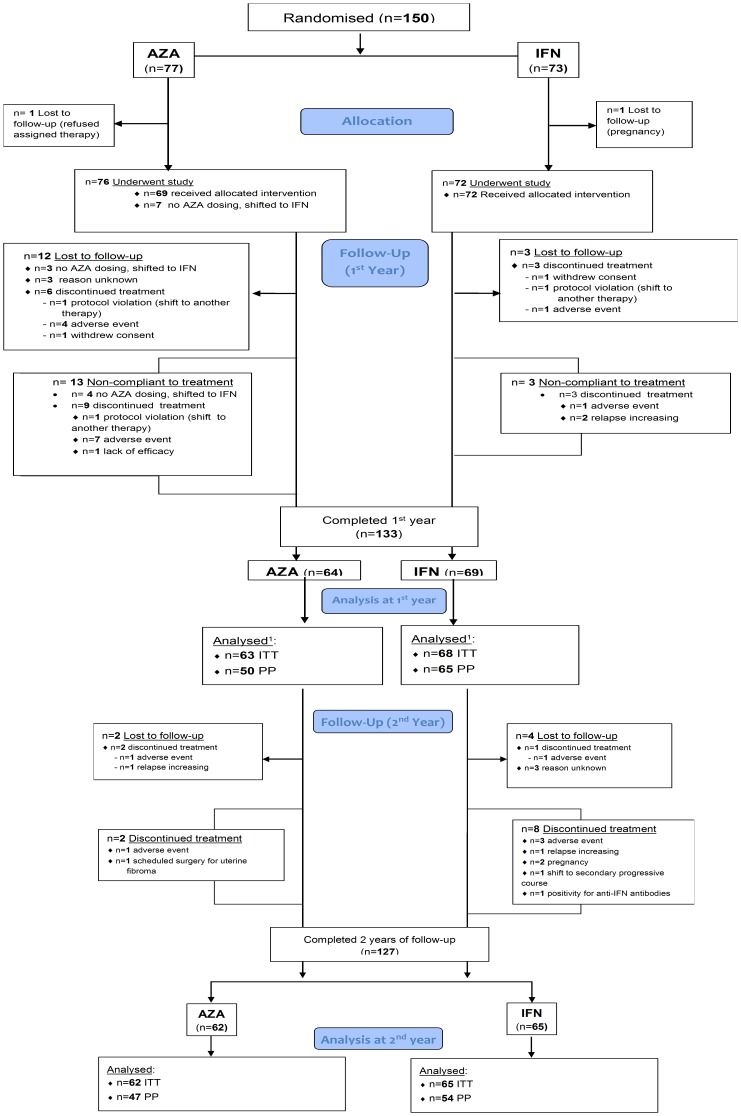
Flow-chart: patient allocation and follow-up. Abbreviations: AZA, azathioprine; IFN, interferon; ITT, intention to treat; PP, per-protocol. ^1^One missing CRF at month 12.

Figure 1 presents patient allocation and follow-up. Of the 150 randomized patients 77 and 73 were AZA- and IFN-assigned respectively. In the IFN group, 26 (36%) were assigned to Avonex, 5 (7%) to Betaferon, 35 (48%) to Rebif 22, and 7 (10%) to Rebif 44. Of the 150 patients screened at baseline, 127 completed the ITT follow-up: 62 (81%) in the AZA group, and 65 (89%) in the IFN group (overall 85%). Eight patients, initially randomized to AZA, refused consent and received IFN (out of these, four were lost to follow up). Including losses to follow up, treatment discontinuations were respectively 30 in the AZA group (39%; with the patients who refused to begin the treatment, n = 8) and 19 in the IFN group (26%). The majority of the discontinuations under AZA occurred in the first year (n = 26; 87%) whereas those under IFN occurred in the second year (n = 12; 63%). The discontinuations were 22 (32%) and 18 (25%) respectively, if only patients who began the treatments are included in the analysis of pharmacological compliance.

Fourteen (47%) of 30 treatment discontinuations in the AZA group and 6 (32%) of 19 discontinuations in the IFN group were due to AEs; 2 (7%) of 30 patients in the AZA group and 3 (16%) of 19 patients in the IFN group discontinued for lack of efficacy. Demographic, clinical characteristics and MRI findings at baseline were highly comparable in both groups (Table 1), even considering the ITT (n = 127), the PP (n = 101), and the MRI (n = 97) populations who completed follow-up [data not shown]. Baseline characteristics were comparable even separately considering patients enrolled during the first and second year of recruitment [data not shown].

**Table 1:** Baseline characteristics of the patients.

Characteristic	AZA (N = 77)	IFN (N = 73)	p-value^1^
Demographic characteristics			
Female – No. (%)	49 (63.6%)	50 (68.5%)	p = 0.53
Age - Years			
Mean ± SD	38.1±8.9	36.6±8.8	p = 0.31
Median (range)	37.9 (21.3–56.5)	37.6 (19.1–58.8)	
Clinical characteristics			
Duration of disease from onset of symptoms - Years			
Mean ± SD	6.8±7.1	5.7±5.7	
Median (range)	3.4 (0.5–25.3)	3.4 (0.3–24.8)	p = 0.53
Relapses in previous 2 years			
Mean ± SD	2.38±0.78	2.41±0.89	
Median (range)	2 (0–5)	2 (0–6)	p = 0.91
No. patients with relapses in previous 2 years - No. (%)			
0–1^2^	3 (3.9%)	2 (2.7%)	
2	48 (62.3%)	47 (64.4%)	p = 0.91
≥3	26 (33.8%)	24 (32.9%)	
No. patients with previous histories of … - No. (%)			
AZA treatment	1 (1.3%)	1 (1.4%)	p = 0.95
IFN treatment	4 (5.2%)	3 (4.1%)	
EDSS score^3^			
Mean ± SD	1.9±0.9	1.9±0.9	
Median (range)	1.5 (1.0–5.5)	1.5 (0.0–5.0)	p = 0.86
Patients with concomitant diseases – No. (%)^4^	5 (6.9%)	4 (5.8%)	p = 0.80

	AZA (N = 61)	IFN (N = 61)	p-value^1^
MRI findings			
Gd+ lesion number			
Mean ± SD	1.64±3.85	2.32±4.53	
Median (range)	0 (0–24)	1 (0–20)	p = 0.38
No. patients with Gd+ lesions - No. (%)			
0	32 (52.5%)	27 (44.3%)	
1–2	20 (32.8%)	23 (37.7%)	p = 0.36
≥3	9 (14.8%)	11 (18.0%)	
T2 lesion load (FLAIR sequences; mm^3^)			
Mean ± SD	15,284±16,466	10,283±11,696	p = 0.16
Median (range)	9,197 (338–73,226)	7,205 (326–61,025)	

Abbreviations: AZA, azathioprine; EDSS, Expanded Disability Status Scale; IFN, interferon; SD, standard deviation.

1 P-values for AZA vs. IFN comparison were obtained through: χ^2^ test with one or two degrees of freedom for sex, number of patients with previous histories of AZA/IFN treatment, number of patients with relapses with concomitant disease and with Gd+ lesions; t-test for age; Mann-Whitney test for duration of disease, number of relapses, EDSS score, number of Gd+ lesions and T2 lesion load.

2 Protocol violations.

3 Scores on the EDSS range from 0 to 10, with higher scores indicating greater degree of disability.

4 The sum does not add up to the total because of some missing values.

### Efficacy - clinical outcomes

**Figure 2:**
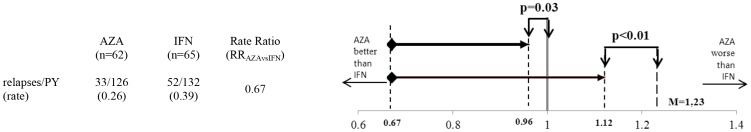
Primary clinical outcome over 2 years: non-inferiority of effect of AZA vs. IFN, represented as annualized relapse rate ratio (RR_AZA/IFN_) compared with the pre-established non-inferiority margin M ( = 1.23) and with a margin M_1_ = 1.0. One-sided 99% CI of the 0.67 ratio (upper-limit, U_L_ = 1.12), represents an effect of AZA vs. IFNs equivalent to at least 75% of the effect of IFNs vs. Placebo. One-sided 95% CI of the same ratio (U_L_ = 0.96), represents an effect of AZA vs. IFNs equivalent to at least 100% of the effect of IFNs vs. Placebo. Abbreviations: AZA, azathioprine; IFN, interferon; PY, person-years; RR, rate ratio.

**Figure 3:**
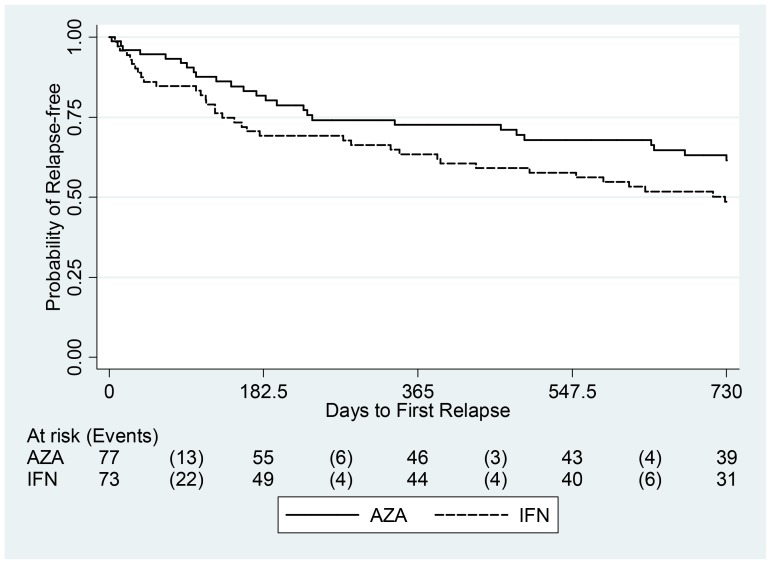
Time to first relapse. Beneath the plot patients at risk and number of events (in brackets) by treatment were reported for each interval of 6 months. Abbreviations: AZA, azathioprine; IFN, interferon.

From the primary efficacy analysis, AZA emerges as significantly non-inferior to IFN (Fig. 2), as the upper limit (U_L_) of the one-sided 95% CI for the annualized relapse RR_AZA/IFN_ was 0.96, i.e., below the non-inferiority margin M ( = 1.23; p<0.01). This U_L_ is also significantly (p = 0.03) below a more stringent non-inferiority margin M1 = 1.0, corresponding to 100% of the effect of IFNs vs. placebo. The U_L_ of the one-sided 99% CI for the RR_AZA/IFN_ (i.e., 1.12), corresponding to the 75% of the IFN effect vs. placebo, was also significantly below the non-inferiority margin of M = 1.23 (p<0.01). The annualized relapse rates observed over two years among the AZA and the IFN treated subjects were 0.26 and 0.39, respectively (p = 0.07, adjusted p = 0.06; Table 2). The corresponding RR_AZA/IFN_ was 0.67 (95% CI, 0.43–1.03) based on the 127 patients who completed follow-up, 0.67 (95% CI, 0.40–1.12) based on 150 randomized patients and using the monotone logistic regression multiple imputation method, and 0.69 (95% CI, 0.43–1.10) using the FCS logistic regression multiple imputation method [data not shown]. Adjusted analysis gave similar results (Table 2), confirming the robustness of the findings. In addition, comparable results were obtained in a sensitivity analysis excluding the Avonex treated patients (the annualized relapse rate over two years among Betaferon or Rebif treated patients was 0.37, with a corresponding RR_AZA/IFN_ of 0.70, 95% CI, 0.43–1.15) [data not shown]. No significant difference was noted between AZA and IFN in the proportion of patients with 0, 1, 2, ≥3 relapses over two years and separately in the first or the second year, the proportion of patients with corticosteroid-treated relapses, and the proportion of patients with no confirmed disability progression over two years. (Table 2). There were six treatment failures in the AZA group and five in the IFN group. For QOL, no difference was observed between the treatments, for both physical and mental-QOL (p = 0.94 and 0.93, respectively) [data not shown]. Figure 3 shows Kaplan-Meier curves of the time to first relapse: no significant difference was observed in terms of log-rank (p = 0.11) or Cox proportional-hazards model results, with a hazard ratio of 0.66 (95% CI, 0.40–1.10). Similar results were obtained in sensitivity analyses excluding Avonex treated patients (log-rank p = 0.15) [data not shown]. The analyses performed in the PP population yielded similar findings [data not shown].

**Table 2:** Secondary clinical outcomes.

Outcome	1^st^ Year			2^nd^ Year			Overall (2 years of follow-up)		
	AZA (N = 63)	IFN (N = 68)	p-value^1^	AZA (N = 62)	IFN (N = 65)	p-value^1^	AZA (N = 62)	IFN (N = 65)	p-value^1^
*Relapses*									
Annualised relapse rate (95% CI)	0.37 (0.25–0.56)	0.47 (0.34–0.67)	p = 0.37	0.18 (0.10–0.32)	0.29 (0.18–0.45)	p = 0.19	0.26 (0.19–0.37)	0.39 (0.30–0.51)	p = 0.07
Adjusted annualised relapse rate (95% CI)^2^	-	-	-	-	-	-	0.27 (0.19–0.38)	0.41 (0.31–0.54)	p = 0.06
No. of patients with relapse - No. (%)									
0	45 (71.4%)	44 (64.7%)	p = 0.63	52 (83.9%)	49 (75.4%)	p = 0.42	39 (62.9%)	31 (47.7%)	p = 0.22
1	14 (22.2%)	17 (25.0%)		9 (14.5%)	13 (20.0%)		15 (24.2%)	23 (35.4%)	
≥2	4 (6.4%)	7 (10.3%)		1 (1.6%)	3 (4.6%)		8 (12.9%)	11 (16.9%)	
No. of patients with relapses treated with corticosteroids – No. (%)									
0	-	-	-	-	-	-	40 (64.5%)	34 (52.3%)	p = 0.22
1	-	-	-	-	-	-	16 (25.8%)	22 (33.9%)	
≥2	-	-	-	-	-	-	6 (9.7%)	9 (13.9%)	
*Disability* 3									
Patients with no confirmed disability progression - % (95% CI)^4^	-	-	-	-	-	-	98.2 (91.5–99.9)	92.0 (81.8–97.4)	p = 0.19
Change from baseline in EDSS score – Mean (95% CI)^5^	-	-	-	-	-	-	−0.08 (−0.31; 0.16)	0.22 (−0.03; 0.47)	p = 0.08

Abbreviations: AZA, azathioprine; IFN, interferon.

1 P-values for AZA vs. IFN comparison were obtained through χ^2^ test with one degree of freedom for rate comparison, χ^2^ test with two degrees of freedom for number of patients with relapses, Fisher's exact test for patients with no confirmed disability progression, and t-test for change in EDSS score.

2 The analyses were adjusted for number of relapses during the previous two years, baseline EDSS score, and duration of disease from symptom onset.

3 The analyses were based on 56 AZA and 50 IFN patients respectively, because of some missing values.

4 A confirmed disability progression was defined as an increase of no less than one point of the EDSS score confirmed at least after six months; 95% CI were estimated through the exact method. All the patients, with the exception of two (who did not report a disability progression), had a baseline EDSS score between 1 and 5.

5 Adjusted for baseline EDSS score.

### Efficacy - MRI outcomes

**Figure 4:**
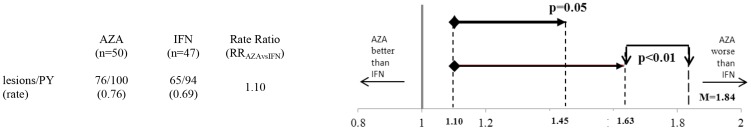
Non-inferiority of the effect AZA vs. IFN on new T2 lesions over 2 years. One-sided 99% CI (upper-limit, U_L_ = 1.63), and one-sided 95% CI (U_L_ = 1.45), of the effect of AZA vs. IFNs as for annualized new T2 lesion rate ratio (RR_AZA/IFN_), compared with the pre-established non-inferiority margin (M = 1.84), representing an effect of AZA vs. IFNs equivalent to the 73% of the effect of IFNs vs placebo. Abbreviations: AZA, azathioprine; IFN, interferon; PY, person-years; RR, rate ratio.

Of the 122 patients given baseline MRI (61 per group), 97 completed the ITT follow-up: 50 (82%) in the AZA group, and 47 (77%) in the IFN group. The ratio of annualized new T2 lesion rates of AZA vs. IFNs was 1.10 (Fig. 4). The corresponding U_L_ of the 95% one-sided CI was 1.45, below the non-inferiority margin M = 1.84, indicating an AZA vs. IFN effect equivalent to at least 73% of the IFNs vs. placebo effect. Moreover, the U_L_ of the one-sided 99% CI for the new T2 lesion RR_AZA/IFN_ (i.e., 1.63) was also significantly below the non-inferiority margin of M = 1.84 (p<0.01). Table 3 summarizes the MRI outcomes: no significant difference was noted between AZA and IFNs for new T2, new CE, and Gd+ lesions. The annualized new T2 lesion rate was 0.69 (95% CI, 0.54–0.88) in the IFN and 0.76 (95% CI, 0.61–0.95) in the AZA patients (p = 0.75). Adjustments for inflammatory activity at baseline, expressed by the Gd+ lesion number confirmed these findings. Analyses performed in the PP population (81 patients: 40 in the AZA and 41 in the IFN group) confirmed these results [data not shown].

**Table 3:** MRI outcomes. New brain lesions.

Outcome	Overall (2 years of follow-up)		
	AZA (N = 50)	IFN (N = 47)	p-value^1^
*New T2 lesions*			
Annualised new T2 lesion rate (95% CI)	0.76 (0.61–0.95)	0.69 (0.54–0.88)	p = 0.75
No. of patients with new T2 lesions - No. (%)			
0	27 (54.0%)	21 (45.0%)	
1–2	11 (22.0%)	18 (38.0%)	p = 0.41
≥3	12 (24.0%)	8 (17.0%)	
*New Combined Unique (CE) lesions*			
Annualised new CE lesion rate (95% CI)	0.78 (0.63–0.98)	0.70 (0.55–0.90)	p = 0.53
*Gd+ lesions*			
Gd+ lesion number			
Mean ± SD	0.20±0.50	0.40±1.35	
Median (range)	0 (0–2)	0 (0–5)	p = 0.52
No. patients with Gd+ lesions - No. (%)			
0	41 (84.0%)	43 (91.5%)	
1–2	8 (16.0%)	1 (2.0%)	p = 0.39
≥3	0 (0.0%)	3(6.5%)	
Missing data	1	0	

Abbreviations: AZA, azathioprine; IFN, interferon.

1 P-values for AZA vs. IFN comparison were obtained through χ^2^ test with one degree of freedom for rate comparison, χ^2^ test with two degrees of freedom for number of patients with lesions, and Mann-Whitney test for Gd+ lesion number.

### Safety comparison

The rate of patients with at least one AE was not different between the two groups (p = 0.28), however the rate of AEs was higher in the AZA group (p<0.01) (Table 4). The most frequently reported AEs were flu-like symptoms, more frequent in IFNs (p<0.01), nausea/vomiting and abnormal blood count more frequent in AZA-treated patients (p<0.01). AE-related discontinued interventions were more frequent among AZA (20.3%) than IFN (7.8%) patients (p = 0.03). SAEs and other AEs are described in Tables S1 and S2 in File S1.

**Table 4:** Adverse Events.

Event	AZA	IFN	p-value^1^
	(N_patients_ = 69, N_events_ = 308, PY = 108)	(N_patients_ = 77, N_events_ = 241, PY = 136)	
**All AEs** 2			
Patients – No./PY and rate (95%CI)	65/108	68/136	p = 0.28
	0.60 (0.47–0.77)	0.50 (0.40–0.64)	
AEs - No./PY and rate (95%CI)	308/108	241/136	p<0.01
	2.85 (2.54–3.19)	1.77 (1.56–2.01)	
**Most frequently reported AEs** 2			
Influenza-like illness			
Patients – No./PY and rate (95%CI)	3/108	39/136	p<0.01
	0.03 (0.01–0.08)	0.29 (0.20–0.39)	
AEs - No./PY and rate (95%CI)	3/108	41/136	p<0.01
	0.03 (0.01–0.08)	0.30 (0.22–0.41)	
Fever			
Patients – No./PY and rate (95%CI)	2/108	19/136	p<0.01
	0.02 (0.00–0.07)	0.14 (0.08–0.22)	
AEs - No./PY and rate (95%CI)	2/108	20/136	p = 0.01
	0.02 (0.00–0.07)	0.15 (0.09–0.23)	
Local allergic reaction			
Patients – No./PY and rate (95%CI)	0/108	13/136	-
		0.10 (0.05–0.16)	
AEs - No./PY and rate (95%CI)	0/108	14/136	-
		0.10 (0.06–0.17)	
Systemic allergic reaction			
Patients – No./PY and rate (95%CI)	3/108	0/136	-
	0.03 (0.01–0.08)		
AEs - No./PY and rate (95%CI)	3/108	0/136	-
	0.03 (0.01–0.08)		
Nausea/vomiting			
Patients – No./PY and rate (95%CI)	30/108	1/136	p<0.01
	0.28 (0.19–0.40)	0.01 (0.00–0.04)	
AEs - No./PY and rate (95%CI)	35/108	1/136	p<0.01
	0.32 (0.23–0.45)	0.01 (0.00–0.04)	
Abnormal blood count			
Patients – No./PY and rate (95%CI)	46/108	24/136	p<0.01
	0.43 (0.31–0.57)	0.18 (0.11–0.26)	
AEs - No./PY and rate (95%CI)	106/108	39/136	p<0.01
	0.98 (0.80–1.19)	0.29 (0.20–0.39)	
Other abnormal blood tests^3^			
Patients – No./PY and rate (95%CI)	24/108	37/136	p = 0.44
	0.22 (0.14–0.33)	0.27 (0.19–0.37)	
AEs - No./PY and rate (95%CI)	46/108	54/136	p = 0.72
	0.43 (0.31–0.57)	0.40 (0.30–0.52)	
Other AE			
Patients – No./PY and rate (95%CI)	51/108	47/136	p = 0.12
	0.47 (0.35–0.62)	0.35 (0.25–0.46)	
AEs - No./PY and rate (95%CI)	70/108	54/136	p<0.01
	0.65 (0.51–0.82)	0.40 (0.30–0.52)	
**Discontinued interventions due to AEs**			
No. of patients with discontinued interventions due to AEs (%)	14 (20.3%)	6 (7.8%)	p = 0.03
**Seriousness of AE** 5			
No. of events (%)^4^			
Minor/Moderate	291 (96.0%)	236 (98.3%)	p = 0.12
Major/Serious	12 (4.0%)	4 (1.7%)	
**Correlation with study treatment**			
No. of events (%)^4^			
Non-correlated/Unlikely	63 (20.7%)	49 (20.4%)	p = 0.95
Possible/Likely	242 (79.3%)	191 (79.6%)	

Abbreviations: AZA, azathioprine; IFN, interferon; PY, person-years.

1 P-values for AZA vs. IFN comparison were obtained through χ^2^ test with one degree of freedom for rate comparison, discontinued interventions due to adverse events, seriousness of adverse event, and correlation of event with treatment.

2 All 95% CI were estimated using the exact method.

3 Liver enzymes, thyroid function and bilirubin level.

4 The sum does not add up to the total because of some missing values.

5 Seriousness judged by the treating neurologist. SAEs classified according to the National Cancer Institute Common Terminology Criteria for AE [21] are reported in Table S1 in File S1.

## Discussion

### Principal findings

This study directly compared AZA and IFN efficacy on clinical and MRI outcomes in relapsing-remitting MS patients. The results indicated that AZA was non-inferior to IFNs in reducing relapses and new brain lesions over two years. The effect size on the primary end point (annualized relapse rate ratio) was 0.67, with the upper CIs indicating that in the worst case scenario efficacy of AZA vs. placebo can be estimated as at least 100% (95% CI) or as at least 75% (99% CI) of that of IFNs, according to the CIs level selected. The effect size on new brain lesions (the main secondary outcome measure) was 1.1 with the upper CI levels (95%) indicating that in the worst case scenario efficacy of AZA vs. placebo could be estimated as at least 73% of that of the IFNs. The direct comparison of AZA and IFN efficacy therefore indicated a similar effect size, in reducing both relapses and new brain lesions. Both treatments were similarly efficacious in time to the first relapse, in slowing disability accumulation, and in the other secondary clinical and MRI outcome measures examined. Both medications showed better efficacy in the second year, probably for a delay in fully exerting their activity during the first months of treatment, at least in part determined by the initial dose titration. The observed lag of efficacy was similar for both treatments.

Similar efficacy of AZA and IFNs was observed both in the ITT and in the PP analysis and in the different sensitivity analyses performed. As in this study the comparator treatment included all the IFNs as a group, a sensitivity analysis excluding Avonex treated patients (probably the less efficacious of the IFNs [30]) confirmed the results of the main analysis.

AZA was compared to all the IFNs as a group because a centralized choice of one specific IFN could have raised allegation of conflict of interests, as in this academically driven independent study the medications were prescribed and charged to the NHS. In addition, under these experimental conditions, a centralized selection of a specific IFN could have reduced and distorted patient accrual in the participating centers.

The remarkable internal consistency between clinical and MRI data, between the ITT and the PP analysis and among the different sensitivity analyses, supported the robustness of the results. It must be pointed out that consistency between ITT and PP analysis is a critical requirement for reliability of non-inferiority studies [23]–[25].

The present study strengthens previous results of AZA vs. placebo [1]–[4] or vs. IFN [14]–[16], and expands previous available data as for the first time MRI was included as an outcome of AZA efficacy, thus allowing contemporary assessment of relapses and brain lesions accumulation. The previous MRI studies [17]–[18] indeed were informative for supporting the hypothesis of AZA efficacy on brain lesions, but were not aimed to assess clinical outcomes and were based on retrospective or open label designs [17]–[18].

It must be noted that the results of the present study were obtained administering AZA at the target dose of 3 mg/Kg/day, adjusted according to leuko/lymphocyte count. This approach was similar to that of the trials that also showed the most remarkable reduction in relapse rates induced by AZA [2]–[4], [15], suggesting that appropriate dosage represents an important variable administering this treatment.

No unknown AEs occurred. Overall similar numbers of patients developed at least one AE. Leuko/lymphopenia in the AZA group was not associated with a higher incidence of infections and should be considered part of the desired mechanism of action. However, treatment discontinuations after AEs were significantly higher in the AZA group, mainly occurring during the first months of treatment. Most of the discontinuations for IFNs were in the second year, confirming already known different temporal AE profile of each treatment.

### Strengths and weaknesses of the study

The main limit of the study was probably the sample size, which resulted smaller than planned. This was due to difficulties in recruiting and retaining patients in the trial, particularly following the change in the Italian NHS reimbursement criteria that occurred during the recruitment period. Indeed, rational basis of a direct comparison and randomization between an old generic medication and a new approved drug were sometimes hard to explain both to neurologists and patients and contributed to these difficulties.

However, the sample size affected only the initial power estimate based on the conservative hypothesis of no difference between the means of the relapse rates. Indeed, the data obtained during the study, showing a difference favoring AZA, allowed a power sufficient to establish non-inferiority at statistically robust levels of significance. Moreover, as documented by Schulz and Grimes [31] trials with low sample size might be acceptable if investigators use methodological rigor to eliminate bias and properly report to avoid misinterpretation.

Another possible limitation could be related to patient knowledge of the treatment. Indeed, out of the patients who refused the assigned treatment, all had been randomized to AZA. As this occurred before the first dose of AZA was administered, it was necessarily due to a different perception by the patients of this therapy with respect to the IFNs, which were specifically approved for MS. Successfully blinding of patients seemed unrealistic given the profoundly different side effects of AZA and IFNs of which the patients had been informed in detail. Indeed, analysis of blinding in previous studies revealed a strong tendency to treatment awareness in patients receiving IFNs [10], [32].

Dropout rates was another possible issue in this study. Although the overall number of patients who withdrew the study was only 15%, a higher number of patients were lost to follow up in the AZA than in the IFN group, mainly during the first year. As this event may have diluted true differences between treatments, sensitivity analyses, based on two multiple imputation methods, were performed and no difference in the RR_AZA/IFN_ estimate was observed, thus confirming the results obtained in the analysis of patients who completed the follow-up.

Finally, the different number of treatment discontinuations observed between the two groups (i.e., 39% of patients on AZA and 26% on IFN) could have impacted the study effect size. However, if only patients who began the treatment according to the study protocol are considered, a similar number of patients discontinued (32% on AZA and 25% on IFNs), suggesting similar compliance of the two medications over two years. The clear difference was that treatment interruptions were more frequent in the first year in the AZA group and in the second year in the IFN group.

### Implications for clinical practice

The present study was the first independent RCT that directly compared efficacy of a generic medication (AZA) to a drug specifically approved for MS (IFN) using a non-inferiority design. The authors believe that the results of this study are robust, clinically meaningful and relevant for clinical practice, supporting AZA as a rational and effective alternative to IFNs in relapsing-remitting MS, particularly considering the convenience of oral administration and the cost, lower than the other available treatments. Nevertheless, the different side effect profiles of both medications have to be taken into account.

## Supporting Information

Checklist S1 CONSORT checklist.
(DOC)

Protocol S1 Trial protocol.
(PDF)

Amendment S1 Amendment to the protocol.
(PDF)

File S1 **Methods S1**, Outcomes. Brain MRI: Scan acquisition specifications. **Table S1**, Serious Adverse Events (SAEs). **Table S2**, AEs – subtypes.
(DOCX)
