# Supplementary material for: Azathioprine versus Beta Interferons for Relapsing-Remitting Multiple Sclerosis: A Multicentre Randomized Non-Inferiority Trial
Source: PLoS One. 2014 Nov 17;9(11):e113371. doi: 10.1371/journal.pone.0113371 (PMC4234663; doi:10.1371/journal.pone.0113371)
Supplement: Protocol S1 — Trial protocol. (PDF) [file pone.0113371.s002.pdf]

STUDY PROTOCOL TO BE SUBMITTED TO THE STUDY SESSION

Institute:

CLINICA NEUROLOGICA, DIPARTIMENTO DI SCIENZE NEUROLOGICHE E DELLA VISIONE, UNIVERSITÀ DI VERONA

1. Proposal Title

MULTICENTER RANDOMIZED CONTROLLED STUDY OF AZATHIOPRINE VERSUS INTERFERON BETA IN RELAPSING-REMITTING MULTIPLE SCLEROSIS

2. Curriculum vitae of the Principal investigator (max 4,000 characters); up to 5 publications (max 5,000 characters)

Principal Investigator

**First Name**	*Clara --- Luca*
**Last name (Family name)**	*Milanese --- Massacesi*
**Curriculum Vitae**	*CLARA MILANESE, born in Legnano (Milano) Italy 1943; Address: IRCCS Istituto Nazionale Neurologico C: Besta, Via veleria 10, Milano Italy . Education: - M.D. Degree, University of Milan 1968;- Specialty in Neurology, University of Pavia 1985; Professional experience: 1968-71 Research Fellow National Neurological Institute C. Besta, Milan; 1971-91 Assistant Neurologist, National Neurological Institute C. Besta, Milan; 1991-present Senior Assistant Neurologist, National Neurological Institute C. Besta, Milan ; 1996-present Head Regional MS Center National Neurological Institute C. Besta, Milan; 2003- present member of the Coordinating Regional Commission for MS. Research activity has been devoted to CNS autoimmune diseases with particular interest in MS. In particular her activity was focused on MS diagnosis (including advanced diagnostic techniques), on MS genetic susceptibility markers and on MS treatment namely on steroids, immuno-suppressive and immunomodulatory drugs efficacy and action mechanisms. She organized and participated in several in international and national collaborative research projects. 1996-1998 A Multicenter RCT on S.C. Interferon B 1a in the treatment of probable MS (ETOMS study). 1996-1999 Meta analysis of clinical trials with Corticosteroids, Cyclophosfamide, Cyclosporin, Interferon beta and Copolymer I in patients affected by MS (It. Health Inst.). 2000-2002 Multicenter RCT to evaluate the efficacy, of oral Glatiramer acetate in RR MS (CORAL study). 2000-2003 Recombinant IFN beta versus Azathioprine in the treatment of RR MS. A multicenter randomized study (It. M. of Health).*<br>*LUCA MASSACESI; Born in Cagliari, 1954,Italy. Address: Department of Neurological and Psychiatric Sciences, Florence University, Viale Morgagni 85, 50134 Florence, Italy.E-mail: massacesi@unifi.it. Position: Full Professor of Neurology. -1982 MD degree, Florence University;- 1982 Stanford University, Ca, USA. “visiting fellow”, Neurology Dept.; - 1984-1988 Research fellow, Dept. Neurology, Florence University; - 1985 “visiting fellow”, Neuroimmunology Branch , NIH, Bethesda, Ma, USA; - 1986 Board Certificate Specialist in Neurology, Florence University; - 1989-1991 Research fellow, Neuroimmunology Unit , Massachusetts General Hospital, Harvard University, Boston, USA; - 1990 Board Certificate Specialist in Clinical Allergy and Immunology , Florence University; - 1991 Staff Neurologist, Department of Neurology, Florence University; - 1993-2001, member of the Scientific Committee of the Italian Multiple Sclerosis Society (AISM); - 1994-1997, coordinator of the “Concerted Action on T-cell Autoimmunity in Multiple Sclerosis” of the European Union (Biomed IV Programme); - 1995-2001 Assistant Professor, Department of Neurology, Florence University; -1997-1999, coordinator for the Multiple Sclerosis Society of Italy (AISM) of a multicentric project for genetic studies on Multiple Sclerosis”; -1999-2004, principal investigator of the study entitled “RCT on the use of different dosages of b IFN1a, also in association with Azathioprine in RRMS patient”; - 1999-2000, member of the Medical Advisory board of the International Federation of Multiple Sclerosis Societies Scientific Programme Committee; - 1998-2005, member of the Executive Committee of the Interdipartimental Magnetic Resonance Center, Florence University; - 1998-2000, member of the “Steering Committee” of the Biomed Programme Project EDMUS Centralised Facility; - 2001-2005, Associated Professor, Department of Neurology, University of Florence, Italy; - 2005, Full Professor, same Department; 1998- to date, chief Tuscany Region Reference Center for Multiple Sclerosis ,Careggi University Hospital.*

**Publication 1**	*Massacesi L, Parigi A, Barilaro A, Repice AM, Pellicanò G, Konze A, Siracusa GF, Taiuti R and Amaducci L. (2005). Efficacy of Azathioprine on Multiple Sclerosis new brain lesions evaluated by Magnetic Resonance Imaging. Arch.Neurol.;62:1843-1847*
**Publication 2**	*Mancardi G.L., Saccardi, R., Filippi, M., Gualandi F., Murialdo A., Inglese M., Marrosu M.G., Meucci G.,Massacesi L., Lugaresi A., Pagliai F., Sormani M.P., Sardanelli F., Marmont A. and the Italian Gitmo-Neuro Intergroup on Autologous Hematopoietic Stem Cell Transplantation for Multiple Sclerosis (2001). Autologous hematopoietic stem cell transplantation suppresses Gadolinium-enhanced MRI activity in multiple sclerosis. Neurology 57:62-68.*
**Publication 3**	*Milanese C, La Mantia L, Salmaggi A Caputo D (2001).Azathioprine and interferon beta-1b treatment in relapsing-remitting multiple sclerosis. J Neurol Neurosurg Psychiatry 70:406-425*
**Publication 4**	*Milanese C, La Mantia L, Salmaggi A, Eoli M (1993). A double blind study on azathioprine efficacy in multiple sclerosis: final report. J Neurol 240:295-298.*
**Publication 5**	*Yudkin PL, Ellison GW, Ghezzi A, Goodkin DE, Hughes RAC, McPherson K, Mertin J, Milanese C.(1991). Overview of azathioprine treatment in multiple sclerosis. The Lancet 338:1051-1055.*

3. Curriculum vitae of the investigators in charge of the units involved in the study; up to 3 publications (max 3,000 characters per curriculum)

Investigator 1

**First Name**	*Graziella*
**Last name (Family name)**	*Filippini*
**Curriculum Vitae**	*Degree 1973 Medical Doctor Degree (cum laude. Università degli Studi di Milano, Italy<br>1977 Specialisation in Neurology (cum laude). Scuola di Specializzazione in Neurologia Università di Parma, Italy<br>1979 Consultant in Neurology. The Italian Health Ministry Rome, Italy<br>1986 Chief Consultant in Neurology The Italian Health Ministry Rome, Italy<br>Professional activity<br>1974- present. Neurologist, National Neurological Institute C. Besta, Milan, Italy<br>1994- present Head of Epidemiology Unit, National Neurological Institute C. Besta Milan, Italy<br>G. Filippini is the head of the Epidemiology and Statistical Unit at the Istituto Neurologico "Carlo Besta" in Milan. She has been working over the last 25 years in the area of research of brain tumors and multiple sclerosis with particular reference to the evaluation of environmental and genetic risk factors. Over the last 10 years she devoted to evaluation of the effectiveness of treatments and, more recently, to evaluation of the accuracy of diagnostic and prognostic tests in multiple sclerosis and brain tumors, with a specific interest in understanding the extent to which research findings can be timely transferred into clinical practice.<br>Dr. Filippini is Author, or co-Author, of over 100 scientific articles published in peer-reviewed journals.<br>Since 1997 Dr. Filippini runs the Multiple Sclerosis Cochrane group part of the International Cochrane Collaboration, an organisation aimed at producing and disseminating the results of systematic reviews of efficacy of health care interventions, and accuracy of diagnostic and prognostic tests. Since 2004 she leads the working group on "Accuracy of diagnostic and prognostic tests" at the Italian Cochrane Center.<br>Dr. Filippini has been Principal Investigator (P.I.) of various grants funded by the Italian National Research Council (CNR), the Italian Ministry of Health, the Associazione Italiana per la Ricerca sul Cancro (AIRC), and the Fondazione Italiana Sclerosi Multipla (FISM).<br>Dr. Filippini is member of the Advisory Boards of: International Society for the Prevention of Tobacco Induced Diseases, Associazione per la Ricerca sull'Efficacia dell'Assistenza Sanitaria (AREAS), Associazione Italiana Neuroepidemiologia, Epidemiologia e Prevenzione, Effective Health Care Italian edition.<br>She is member of the Brain Tumor Epidemiology Consortium (BTEC) whose goal is to establish multi-centered collaborations that will lead to a better understanding of the causes and risk factors involved in the development of pediatric and adult brain tumors. The Central Brain Tumor Registry of the United States (CBTRUS) provides BTEC with business support.<br>Dr. Filippini has been: Visiting Professor at the National Health Institute in Rome (1984 and 1990), Senior Research Associate in Epidemiology and Biostatistics at the European Institute of Oncology (Milan, Italy) from 1994 to 2002.<br>She is teachi...*
**Publication 1**	*Sellebjerg F, Barnes D,Filippini G,Midgard R,Montalban X,Rieckmann P,Selmaj K,Visser LH,Sorensen PS. EFNS guideline on treatment of multiple sclerosis relapses: report of an EFNS task force on treatment of multiple sclerosis relapses. Eur J Neurol 2005;12:939-46*

**Publication 2**	*Munari LM,Filippini G Lack of evidence for use of glatiramer acetate in multiple sclerosis. Lancet Neurol 2004; 3: 641-2*
**Publication 3**	*Filippini G,Munari L,Incorvaia B,Ebers GC,Polman C,D'Amico R,Rice GP. Interferons in relapsing remitting multiple sclerosis: a systematic review. Lancet 2003;361:545-52*

Investigator 2

**First Name**	*Alessandra*
**Last name (Family name)**	*Solari*
**Curriculum Vitae**	*Born in Borgotaro (Parma) 25 March 1957*<br><br>*POSITION/CONTACT INFO*<br>*Senior Neurologist Epidemiology Unit*<br>*National Neurological Institute C. Besta*<br>*Phone: +39 02 2394 2391*<br>*Email: solari@istituto-besta.it*<br><br>*EDUCATION/TRAINING*<br>*1981- Degree in Medicine, Parma University (summa cum laude)*<br>*1984- Board in Neurology, Parma University (summa cum laude)*<br>*1978/1981- Internship at Clinical Neurology Dept, Parma University*<br>*1984/1989- Internship at C. Besta Nat. Neurol. Inst.Milan*<br>*1985 -Eighth Residential Course on Statistical Methods for Biological Researchers, Biometric Society Italy*<br>*1990/1994- Research period Italian Nat. Research Council, ITBA Milan*<br>*1997- Residential Course on Medical Statistics & Clinical Epidemiology, Italian Nat. Research Council & Harvard School of Public Health*<br>*2005- Degree of Hospital Managings; Inst. of Economics, Administration and Management, Milan BicoccaUniversity*<br><br>*RESEARCH INTERESTS*<br>*Dr. Solari's main area of research is validation of instruments and outcome measures (particularly patient-reported) for clinical, epidemiologic, and quality of care studies in neurological diseases, particularly multiple sclerosis (MS). Other main interest is design and conduction of independent RCTs on rare diseases, and non-pharmacological interventions.*<br><br>*Healt-Related Quality of Life (HRQOL), Patient Satisfaction & Clinical Decision Making*<br>*Dr. Solari is carrying out a community-based prospective study on people with MS, examining relationships between disease status, HRQOL and health care utilization (Italian MS Society). She is devising a structured information aid for newly-diagnosed MS (US National MS Society PP1220).*<br>*She validated the following: A patient self-assessed version of the Minimal Record of Disability for MS; The Italian version of the MSQOL-54(the most-used disease-specific HRQOL questionnaire) (Italian MS Society; Italian Ministry of Health); The Italian version of the Chicago Multiscale Depression Inventory (Italian MS Society).*<br><br>*RCTs*<br>*Dr. Solari devised the first RCT on efficacy of physiotherapy in MS people (Italian MS Society; Italian Ministry of Health);Chief investigator in the CRIMS Trial (multicenter double-blind RCT on computer-aided retraining of memory & attention in MS. US National MS Society, PP079); Co-chief investigator in the following: FIRST Trial (multicentre RCT on antiepileptic treatment at first tonic-clonic seizure); Placebo-controlled double-blind RCT on idebenone in Friedreich's ataxia; SMART trial (multicentre RCT on the efficacy of gabapentin in spinal muscular atrophy. Telethon Italy). Dr. Solari performed data analysis in the SMA-Trip trial (placebo-controlled RCT on efficacy of 4-phenylbutyrate in spinal muscular atrophy). Co-chief investigator & trial coordinator of the ongoing CMT-TRIAAL (multicentre double-blind placebo-controlled RCT of ascorbic acid treatment in Charcot-Marie-Tooth 1a disease. Telethon Italy).*
**Publication 1**	*R Saccardi, G Mancardi, A Solari, A Bosi, P Bruzzi, P Di Bartolomeo, A Donelli, M Filippi, A Guerrasio, F Gualandi, G La Nasa, A Murialdo, F Pagliai, F Papineschi, B Scappini, and A M Marmont, on behalf of the Italian GITMO-Neuro Intergroup. Autologous HSCT for severe progressive Multiple Sclerosis in a multicenter trial: impact on disease activity and quality of life. Blood 2005;105(6):2601-2607.*
**Publication 2**	*Mariotti C, Solari A, Torta D, Marano L, Fiorentini C, Di Donato S. Idebenone treatment in Friedreich patients: one-year-long randomized placebo-controlled trial. Neurology 2003;60(10):1676-1679*
**Publication 3**	*Solari A, Filippini G, Gasco P, Colla L, Salmaggi A, La Mantia L, Farinotti M, Eoli M, Mendozzi L. Physical rehabilitation has a positive effect on disability in multiple sclerosis patients. Neurology 1999;52:57-62*

Investigator 3

**First Name**	*Maria Donata*
**Last name (Family name)**	*Benedetti*
**Curriculum Vitae**	*Maria Donata Benedetti, born in Rovereto (Trento) 12 May 1955*<br><br>*Education and Training*<br>*1980 Medical Graduation, Medical School of Padua in Verona (with honour)*<br>*1984 Specialization in Neurology, University of Verona (with honour)*<br>*1986 Master of Science in Epidemiology, School of Public Health, Columbia University, New York*<br>*1990 Ph.D. in Neurological Sciences, University of Verona*<br>*1998 Visiting Scientist, Department of Health Sciences, Mayo Clinic, Rochester, Minnesota, U.S.A.*<br><br>*Present Position: Clinical Senior Assistant, Institute of Neurology, Azienda Universitaria-Ospedaliera,Verona*<br><br>*Principal research interests: Neuroepidemiology, Multiple Sclerosis*<br><br>*Since 1994 dr. Benedetti is teacher of Neuroepidemiology and Biostatistics in the School of Neurology, University of Verona. She obtained several grants for Health researchs: from Veneto Region (1994,Prevalence of dementia in and disability in a sample of elderly in Verona; 1998, Delay in the hospital*<br>*admission of patients with acute stroke in Verona) and from F.I.S.M. (2001, Epidemiologic and genetic study on Multiple Sclerosis in Verona province).*<br><br>*Since 1999 she is responsible of the Multiple Sclerosis outpatients centre of the Institute of Neurology, Verona.*<br><br>*Dr. Benedetti is member of the scientific board of the Italian Association of Neuroepidemiology*
**Publication 1**	*Gomez-Lira M, Moretto G, Bonamini D, Benedetti MD. Pignatti PF, Rizzuto N, Salviati A.*<br>*Myelin-oligodendrocyte glycoprotein polymorphisms and multiple sclerosis. J Neuroimmunol 2002, 133:241-243*
**Publication 2**	*Benedetti MD, Salviati A, Filipponi S, Manfredi M, De Togni L, Gomez-Lira M, Stenta G, Fincati E, Pampanin M, Rizzuto N, Danti G. Prevalence of dementia and apolipoprotein E genotype distribution in the elderly of Buttapietra, Verona Province, Italy. Neuroepidemiology 2002,21:74-80*
**Publication 3**	*Benedetti MD, Bower JH, Maraganore DM, McDonnell SK, Rocca WA. Smoking, alcohol, and coffee consumption and Parkinson's disease: a population-based case-control study. Neurology 2000, 55:1350-1358*

Investigator 4

**First Name**	*Antonio*
**Last name (Family name)**	*Bertolotto*
**Curriculum Vitae**	*Antonio Bertolotto, MD, Professional experience and education*<br><br>*Antonio Bertolotto, graduated in Medicine in 1977 and specialized in Neurology in 1981 at the University of Turin. He worked as Clinical Neurologist in the University of Turin from 1981 to1992, then he moved to the new Center of the University of Turin at S. Luigi Hospital, Orbassano (Turin), where he created the Multiple Sclerosis Center and Clinical Neurobiology Laboratory.*<br>*Dr. Bertolotto is involved in both clinical activity and research in MS: his daily activity includes diagnosis and treatment of MS patients, as the MS Center at Orbassano is now the Regional Referral MS Center in Piedmont (more than 4 million inhabitants).*<br>*The Clinical Neurobiology Laboratory is a component of the European network for anti-IFNbeta antibodies (NABIMS) and it serves as the Italy center for the detection of antibodies neutralizing Interferon beta.*<br>*The research at the MS center and Clinical Neurobiology Laboratory is focused on the detection of Interferon beta non responders, in the Italian hematopoietic stem cell transplantation project and in several other clinical trials.*<br>*Dr. Bertolotto is a member of the scientific board of the Italian Association for MS and of the "Therapeutics and Technology Assessment subcommittee of the American Academy of Neurology" for Interferon beta Neutralizing antibodies.*<br><br>*Dott. Antonio Bertolotto*

	Orbassano, January 2006
**Publication 1**	*Bertolotto A, Sala A, Malucchi S, Marnetto F, Caldano M, Di Sapio A, Capobianco M, Gilli F. Biological activity of interferon betas in patients with multiple sclerosis is affected by treatment regimen and neutralization antibodies.J Neurol Neurosurg Psychiatry 75: 1294-1299, 2004*
**Publication 2**	*Malucchi S, Sala A, Gilli F, Bottero R, Di Sapio A, Capobianco M, Bertolotto A. Neutralizing antibodies reduce the efficacy of beta IFN during treatment of multiple sclerosis. Neurology 62: 2031-2037, 2004*
**Publication 3**	*Gilli F, Marnetto F, Caldano M, Sala A, Malucchi S, Di Sapio A, Capobianco M, Bertolotto A. Biological responsiveness to first injection of interferon-beta in patients with multiple sclerosis. J Neuroimmunol 158: 195-203, 2005*

Investigator 5

**First Name**	*Giovanni Luigi*
**Last name (Family name)**	*Mancardi*
**Curriculum Vitae**	*Graduated in Medicine in 1972 at the University of Genova, became specialist in Neurology in 1976 and in Neurological Rehabilitation in 1978. He works in the Department of Neuroscience, Ophthalmology and Genetic of the University of Genova, Italy. He was educated in Neuropathology at the laboratory of Neuropathology of the Institute of Clinical Neurology of Genova and at the University of Cincinnati, Usa, where he worked during 1980/81 as research fellow. He is member of the Italian Neurological Society, the Italian Association of Neuropathology and the Association of Neuroimmunology. He has been in 1997-2001 “Secretary” of the study group of peripheral nerve disorders. Member of the editorial Board of “Neurological Sciences”. He has been in 2003 President of the Italian Association of Neuropathology. He has been “Secretary” of the Italian Neurological Society from 2001 to 2005. Since 2005, he is “Tresaurer” of the Italian Neurological Society. He has been designed on October 10 2001 by the Faculty of Medicine of University of Genova as full professor of Neurology. He is Director of the II Neurological Clinic of the University of Genoa. President from 2003 of the Scientific Committee of the Italian Multiple Sclerosis Association. His scientific activity is documented by 157 papers published in journals quoted by ISI.*
**Publication 1**	*Saccardi R, Mancardi GL, Solari A et al. Autologous HSCT for severe progressive multiple sclerosis in a multicenter trial: impact on disease activity and quality of life. Blood. 2005;105:2601-7.*
**Publication 2**	*Inglese M, Mancardi GL, Pagani E, Rocca MA, Murialdo A, Saccardi R, Comi G, Filippi M; Italian GITMO-NEURO Group on Autologous Hematopoietic Stem Cell Transplantation. Brain tissue loss occurs after suppression of enhancement in patients with multiple sclerosis treated with autologous haematopoietic stem cell transplantation. J Neurol Neurosurg Psychiatry 2004;75:643-4.*
**Publication 3**	*Solaro C, Brichetto G, Amato MP, Cocco E, Colombo B, D'Aleo G, Gasperini C, Ghezzi A, Martinelli V, Milanese C, Patti F, Trojano M, Verdun E, Mancardi GL; PaIMS Study Group. The prevalence of pain in multiple sclerosis: a multicenter cross-sectional study. Neurology. 200;63:919-21.*

Investigator 6

**First Name**	*Carlo*
**Last name (Family name)**	*Pozzilli*
**Curriculum Vitae**	*Professor Pozzilli was born in Rome, Italy, in 1954. He studied at the University of Rome "La Sapienza", from which he obtained his medical degree in 1979. He then moved to the Hammersmith Hospital in London, UK, to take up a research post. In 1983, he became a Board Certified Neurologist at the University of Rome, and subsequently, in 1986, a Research Fellow in the Department of Radiology, Tohoku University, Japan.<br>In 1987, he was awarded a PhD in Clinical Neurosciences by the University of Rome, and continued as Assistant Professor of Clinical Neurology at the University of Rome, and then again to Associate Professor of Clinical Neurology, in 2000.<br>In 2002 he became Chief of the Multiple Sclerosis Center of Ospedale S.Andrea,University of Rome.<br>Professor Pozzilli's fields of interest include the clinical aspects and treatment of multiple sclerosis, neuroimaging and neuroimmunology; he has published nearly 140 papers and reviews on these subjects. Professor Pozzilli is a member of the European Committee for Treatment and Research in Multiple Sclerosis, the ItalianNeurological Society, and the International Federation of Multiple Sclerosis (International Medical Advisory Board). He partecipated as a first investigator or in the Steering Committe of several multicentre clinical trials on patients*

	with Multiple Sclerosis.
**Publication 1**	*Interferon beta-1b in secondary progressive MS: a combined analysis of the two trials.Kappos L, Weinshenker B, Pozzilli C,et al. Interferon beta-1b in Secondary Progressive Multiple Sclerosis Trial Steering Committee and Independent Advisory Board.Neurology. 2004 23;63:1779.*
**Publication 2**	*Hommes OR, Sorensen PS, Fazekas F, Enriquez MM, Koelmel HW, Fernandez O, Pozzilli C, O'Connor P.Intravenous immunoglobulin in secondary progressive multiple sclerosis: randomised placebo-controlled trial. Lancet. 2004;364:1149-56.*
**Publication 3**	*Tomassini V, Paolillo A, Russo P, Giugni E, Prosperini L, Gasperini C, Antonelli G, Bastianello S, Pozzilli C. Predictors of long-term clinical response to interferon beta therapy in relapsing multiple sclerosis. J Neurol. 2005 Sep 14;*

Investigator 7

**First Name**	*Maria Rosaria*
**Last name (Family name)**	*Tola*
**Curriculum Vitae**	*MARIA ROSARIA TOLA, nata a Orune (Nuoro) il 4/2/1948, laureata in MEDICINA E CHIRURGIA nel 1973 presso l'Università di Sassari. Specialista in Neurologia. Regolarmente iscritta all'albo professionale dei Medici Chirurghi della provincia di Ferrara.*<br>*Vincitrice di BORSA DI STUDIO MINISTERIALE , negli anni accademici 1973-74 e 1974-75.*<br>*Titolare di CONTRATTO QUADRIENNALE dal 1/8/1975e dal 1/8/1976.*<br>*Dal 1/8/1980, qualifica i RICERCATORE CONFERMATO presso l'Istituto di Clinica Neurologica dell'Università di Ferrara.*<br>*Professore Associato per la Disciplina "Neurologia" con decorrenza dal 31/01/1985.*<br>*Dal 31/12 1992 ottiene l'affidamento della responsabilità del Modulo Funzionale di "Neurochimica e Neuroimmunologia" tipo B.*<br>*Dal 1997 al 2002 Presidente del Centro di Servizio e Studi della Sclerosi Multipla e delle Malattie Demielinizzanti.*<br>*A decorrere dal 1/5/1999 conferimento di incarico quinquennale di Dirigente Medico di II livello di Neurologia; a partire da tale data è collocata in aspettativa dall'Università.*<br>*Collabora alla promozione del centro di studi della Malattia di Alzheimer , istituito con un accordo di programma tra Comune , Università degli Studi, Casa di Riposo per Anziani, Azienda Ospedaliera "Arcispedale S.Anna" e Azienda USL di Ferrara e dal 28 agosto 2000 viene nominata responsabile del Centro Esperto per le Demenze.*<br>*Con delibera del 7 luglio 2000 del Direttore Generale dell'Azienda Ospedaliera-Universitaria viene nominata Direttore del Dipartimento Misto di Neuroscienze applicate alla clinica a decorrere dal 1 settembre 2000.*<br>*E' titolare di insegnamento presso diverse scuole di specializzazione dell'Università di Ferrara.*<br>*Ha sempre svolto attività assistenziale a tempo pieno, in regime di convenzione fino all'assunzione dell'incarico di Dirigente di II Livello.*<br>*La sua attività scientifica è rappresentata da oltre 250 PUBBLICAZIONI su riviste italiane e straniere, molte delle quali censite dal current index, 15 capitoli di libri.*
**Publication 1**	
**Publication 2**	
**Publication 3**	

Investigator 8

**First Name**	*Gioacchino*
**Last name (Family name)**	*Tedeschi*
**Curriculum Vitae**	*GIOACCHINO TEDESCHI . Birth: 1952, Benevento, Italy; Address: Second Department of Neurology, Second University of Naples (SUN), Piazza Miraglia 2, Naples 80138, Italy. Phone 081 5665095; Fax 081 5665096; gioacchino.tedeschi@unina2.it*<br>*1977, Medical Degree, Faculty of Medicine, University of Naples (Italy)*<br>*1981, Board Certified in Neurology, Faculty of Medicine, University of Naples*

	1979-80 Visiting Scientist, Dept. of Clinical Research, SYNTHELABO Research Institute, Paris<br>*1980&#64979;82, Research Assistant, Dept of Pharmacology and Therapeutics University of Wales, Cardiff (U.K.)*<br>*1984&#64979;9, Assistant Professor of Neurology, University of Naples*<br>*1993-96, Visiting Scientist, Neuroimaging Branch, NINDS, NIH, Bethesda*<br>*1992-00,Associate Professor of Neurology, Faculty of Medicine, SUN*<br>*2000-to date Full Professor of Neurology, Faculty of Medicine, SUN*<br>*2001-to date Chief Department of Neurology, SUN*<br>*2003-to date Chief Neuroimaging Study Group, Italian Society of Neurology*<br>*2004-to date Secretary Italian Society of Neurology*
**Publication 1**	*Tedeschi G, Lavorgna L, Russo P, et al. Brain atrophy and lesion load in a large population of patients with multiple sclerosis.Neurology. 2005 26;65:280.*
**Publication 2**	*Tedeschi G and Gallo P. (2005). Multiple sclerosis patients and immunomodulation therapies: the potential role of new MRI techniques to assess responders versus non-responders. Neurol Sci.;26 Suppl 4:s209-12.*
**Publication 3**	*Savettieri G, Messina D, Andreoli V, Bonavita S, Caltagirone C, Cittadella R, Farina D, Fazio MC, Girlanda P, Le Pira F, Liguori M, Lugaresi A, Nocentini U, Reggio A, Salemi G, Tedeschi G, Trojano M, Valentino P, Quattrone A. Gender-related effect of clinical and genetic variables on the cognitive impairment in multiple sclerosis. J Neurol. 2004 Oct;251(10):1208-14.*

Investigator 9

**First Name**	*Salvatore*
**Last name (Family name)**	*Amoroso*
**Curriculum Vitae**	*Date and place of birth: August 28,1953, Cosenza Italy.*<br>*Address: Department of Neuroscience, Università Politecnica of Ancona, Via Tronto 10/a, 60020 Ancona, Italy.E-mail: s.amoroso@univpm.it , Current position: Full Professor of Pharmacology, Medical School University of Ancona 2001 ...; Associate Professor Cell. and Mol. Pharmacology, Univ. Naples "Federico II" 1992-2001. Tutor of PhD in Neuropsicopharmacology and Toxicology Univ. Naples "Federico II". Member of the Italian Society of Pharmacology.Member of the Italian Society of Toxicology. Member of the Italia society of Neuroscience. Ministry of Health "Communities Medicine" Fellowship 1982. French Foreign Office Fellowship 1988. EMBO Fellowship 1989. C.N.R.S. French Fellowship 1990. EMBO Fellowship 1994. Referee J.Neurochem. Referee J.Neurosci.Res. Referee Pharmacol.Res. Foreign stages at Dept.Biochem. Univ. Nizza, 1988-1989; CNRS, Cell. Mol.Pharmacol Dept., Sophia-Antipolis (Valbonne), France. Prof. M.Lazdunski 1989-1990. Dept of Biochem.Univ. Dundee, Scotland Prof. D.G.Nicholls 1994 Director of the project on "Pharmacoeconomy" Hospital-University, Ancona 2001. Research Project:"Altri interventi" CNR:Evaluation of mechanisms involved in excytotoxic aminoacid release induced by ischemia and/or hypoxia. CNR (Italy)Genic expression of glutamate receptors in cerebral ischemic models 1995. MURST 40%: new evaluation approaches in toxicology 1995-1996. Istituto Superiore Sanità: Chemical-Physical properties of drugs and their safety. CNR 1997-1998: Strategic Project on environment. CNR 1997: Sciences and Technologies of environment and Habitat. Ministero Sanità: Physiopathology of ischemic cerebral damage 1998. CNR CT04: Role of NO on sodium-calcium exchanger activity during ischemic conditions. 1999. Regione Campania. POP 1999. PRIN 2003 (MIUR). Fondazione Cariverona 2003-2004.*
**Publication 1**	*Secondo A, Sirabella R, Formisano L, D'Alessio A, Castaldo P, Amoroso S, Ingleton P, Di Renzo G, Annunziato L. Involvement of PI3'-K, mitogen-activated protein kinase and protein kinase B in the up-regulation of the expression of nNOSalpha and nNOSbeta splicing variants induced by PRL-receptor activation in GH3 cells. J Neurochem. 2003 Mar;84(6):1367-77.*
**Publication 2**	*Tortiglione A, Pignataro G, Minale M, Secondo A, Scorziello A, Di Renzo GF, Amoroso S, Caliendo G, Santagada V, Annunziato L. Na+/Ca2+ exchanger in Na+ efflux-Ca2+ influx mode of operation exerts a neuroprotective role in cellular models of in vitro anoxia and in vivo cerebral ischemia. Ann N Y Acad Sci. 2002 Nov;976:408-12*
**Publication 3**	*Tortiglione A, Minale M, Pignataro G, Amoroso S, DiRenzo G, Annunziato L. The 2-oxopyrrolidinacetamide piracetam reduces infarct brain volume induced by permanent middle cerebral artery occlusion in male rats. Neuropharmacology. 2002 Sep;43(3):427-33*

Investigator 10

**First Name**	*Edo*
**Last name (Family name)**	*Bottacchi*
**Curriculum Vitae**	*Edo Bottacchi was born in Cannero Riviera ,the 31 of July, 1951.<br>Graduated with Honours at the University of Milan Medical School.<br>Internship at the National Neurological Institute "Carlo Besta" in Milan from 1977 to 1982.<br>In July 1981 he obtained the Speciality Certification in Neurology and in 1985 in Neuropathology at the University of Milan.<br>In this first period the main interest was in biochemistry of neuro-degenerative Disorders of CNS.<br>In 1983 was vice-head in Department of Neurology Regional Hospital of Aosta Valley.<br>The main clinical interest was in the following topics: Epilepsy, Neuropathy and Neurophisiology.<br>From 1987 to 2005 was Director of Neurology and Neurophisiology Department of Regional Hospital of Aosta Valley.<br>He interest gradually focused on Stroke as both clinical and epidemiological aspects.<br>In march 2003 he was called to cover the position of Director of "Dipartimento delle Medicine Specialistiche".<br>Member of several Neurological Societies he published over 100 papers, many on International Journals, and over 200 presentations at National and International Meetings.<br><br>Aosta: 24-6-05 Dr Edo Bottacchi*
**Publication 1**	*C. Milanese, L. La Mantia, R.Palumbo, V.Martinelli, A. Murialdo, M. Zaffaroni, D. Caputo, R.Capra, R. Bergamaschi, the North Italy Multiple Sclerosis Group*: A post-marketing study on interferon B 1b and 1a treatment in<br>relapsing-remitting multiple sclerosis: different response in drop-outs and treated patients.J Neurol Neurosurg Psychiatry 2003;74:1689-1692.*
**Publication 2**	*G. Corso, G. Giardini, C. Lia, M. Di Giovanni, M. Di Benedetto, M. Pesenti-Campagnoni E. Bottacchi.<br>C-Reactive protein, acute phase of ischemic Stroke and outcome a three months : the data of Aosta Valley Stroke registry. Cerebrovascular Diseases 2005; 19(S2):146.*
**Publication 3**	*W Hacke, M Kaste, C Fieschi, R von Kummer, A Davalos, V Laurre, E Bluhmki et al. for the Second European-Australasian Acute Stroke Study. Randomiset double-blind placebo-controlled trial of Thrombolytic therapy with intravenous alteplase in acute ischaemic stroke (ECASS II). Lancet,1998;352:1245-1251.*

Investigator 11

**First Name**	*Giorgio*
**Last name (Family name)**	*Giuliani*
**Curriculum Vitae**	*GIORGIO GIULIANI - M.D.<br>Born the 20th of May 1948, graduate in Medicine and Surgery in 1972 at the University in Perugia. Specialised in Neurology at the University of "Sacro Cuore" in Rome and in Infantile Neuropsychiatry at the University of Pisa. He carries on a didactic activity in the limit of the university with several rule, graduate employed to exercitations and teacher at the Specialization School in Neurology from '77 until now. Member of some Scientific Societies, particularly promoter member of the Neuroepidemiology Section of the S.I.N.<br>Activities for scientific research. In the past he developed researches about neurophysiopatology and epileptology and he participated in several studies about antiepileptic drugs. Since 1980 he was starting research clinical epidemiology; particularly he developed a methodology of investigation based on the analysis of "target" drugs utilization for neurological diseases. Other major subject of his research is the Multiple Sclerosis clinical and epidemiological aspects, other topics were concerning about the utilization of CT (Computerized Tomography) and MRI (Magnetic Resonance Imaging) in Neurology.<br>In the end he participated to multicentric studies about Multiple Sclerosis (epidemiological, clinical, Quality of Life, organizational issues, etc), to several systematic revisions about this disease in the Cochrane Collaboration Library and promoted and carried out studies on the disease in the Marche Region. On the whole he is author , alone or in collaboration, of about one hundred and twenty medical publications.*
**Publication 1**	*C Taus, B. Viti, L. De Dominicis, A. Fiè, G. Giuliani : Valutazione della tollerabilità di diversi interferoni beta nella terapia della Sclerosi Multipla, Riv. Neurobiologia, 43, (5), 379-384, 1997*
**Publication**	*G. Giuliani, C Taus: La Cochrane Collaboration: storia, obiettivi ed attività italiane in ambito neurologico, Riv.*

**2**	*Neurobiologia, 46, (1/2), 13-20, 2000*
**Publication 3**	*E. Pucci, E. Cartechini, C Taus and G. Giuliani: Why physicians need to look more closely at the use of complementary and alternative medicine by multiple sclerosis patients, European Journal of Neurology, 11:263-267, 2004*

4. Abstract

BACKGROUND: Interferon beta (IFNB) is currently considered the first line treatment for Relapsing-Remitting Multiple Sclerosis (RRMS), but in many patients this drug shows no or little efficacy or is not well tolerated. In addition IFNB costs are relevant for the Italian National Health System. Azathioprine is an immunosuppressive drug, well tolerated and unexpensive, used since many years in autoimmune diseases, but whose efficacy in MS has been generally considered marginal, although it has never been studied with modern methodologies in large RCTs. Nonetheless, several studies on small samples of MS patients and recent post-marketing metanalyses suggest that it is probably as effective as IFNB on clinical and Magnetic Resonance Imaging (MRI) outcomes measures of disease activity.
OBJECTIVES: To direcly compare in patients with RRMS: - azathioprine to IFNB efficacy in modifying disease activity; - safety profile of the two medications and their impact on quality of life.
METHODS: multicentric, randomized, single-masked, actively controlled, non-inferiority study; 350 RRMS outpatients from 35 italian MS centres, age 18-55 years, with at least two clinical relapses in the preceding 2 years and Expanded Disability Status Scale (EDSS) of 1.0-5.5, randomly assigned to two arms of treatment: azathioprine (3 mg/kg/day) or IFNB (any of the formulations approved for RRMS therapy). The study will last 2 years. Primary outcome measure will be the number of relapses per patients over the study period. Secondary outcomes will be: number and volumes of new brain lesions, change of total brain lesion load, changes of disability at 2 years; the safety profile of the two treatments in terms of frequency and severity of adverse events and impact on quality of life. All patients will receive neurologic examination every third month and brain MRI, yearly. Analysis will be carried out both by intention to treat and by protocol. Noteworthy, this will be the first study to assess MRI and clinical outcomes in a large sample of RRMS patients treated with azathioprine versus IFNB.
EXPECTED RESULTS: similar efficacy on clinical outcomes and on brain lesions is expected for both the medications; a better safety profile and impact on quality of life of azathioprine is expected.

5. Running title

AZATHIOPRINE VS INTERFERON BETA IN RRMS

6. Key words

N.	Key words
1.	*AZATHIOPRINE*
2.	*IFNB*
3.	*MULTIPLE SCLEROSIS*
4.	*RCT*
5.	*RELAPSES*

7. Background and rationale

7.1. Eziopathogenesis of Multiple Sclerosis (MS).
MS is an inflammatory disease affecting mainly the Central Nervous System (CNS) white matter with a very high socioeconomic impact, being the most frequent nontraumatic cause of disability in young adults. Autoimmune pathogenetic mechanisms are supposed to be involved in the development of MS lesions, however the etiology of the disease is still unknown and no curative therapies are available.
7.2. Interferon beta (IFNB) in MS.
IFNB at various doses and by different routes of administration is worldwide approved since more than ten years for the treatment of Relapsing-Remitting MS (RRMS). The biological mechanisms of IFNB effects in MS are controversial, but they probably involve immunoregulation by suppressor T-cells activation and proliferation, and inhibition of T-cells ability to cross the blood-brain barrier and enter CNS. The first IFNB approved for the treatment of MS (1993 in U.S. and 1996 in Italy) was IFNB-1b (Betaseron®). At the high dose tested, 8 M IU subcutaneous (s.c.) on alternate days, IFNB-1b showed a 33% decrease in the relapse rate against placebo at 2 years in a randomised, placebo-controlled study (RCT) involving 372 RRMS patients (IFNB Study Group, 1993). The final results at 5 years, however, raised some concern about the large number of dropouts at 5 years (more than 40%), the lack of statistically significant reduction in exacerbation rate after the second year, the high proportion of subjects (38%) developing neutralizing antibodies to IFNB (NAB), and the absence of effect on disability progression (secondary end-point) measured on the EDSS (Kurtzke, 1983) (IFNB Study Group, 1995). IFNB effects on MRI outcomes, however, were more evident, showing a highly significant reduction in the number of new T2 lesions (-60%) and in the increase of brain lesion burden compared with placebo (Paty et al., 1993). In a second RCT 301 RRMS patients were treated with weekly intramuscolar (i.m.) injections of IFNB-1a 30 μg Avonex®) (Jacobs et al., 1996). This study was prematurely terminated for statistical reasons, although only 57% of patients completed 2 years of follow-up. In this study a significant delay in time to sustained progression of at least 1 point on the EDSS in treated group (22%) against placebo (35%) was observed, whereas the effect on relapses reduction was barely significant (18%). IFNB-1a i.m. activity on new T2 lesions reduction was of 33%. IFNB-1a i.m. was approved for the treatment of RRMS (1996 in U.S. and 1997 in Italy). Later on IFNB-1a by s.c. route three times weekly (Rebif®) was also approved after a RCT on 560 RRMS

patients showed at 2 years a relapse rate reduction similar to that of IFNB-1b: 27% for the 22 µg arm and 32% for the 44 µg arm against placebo (PRISMS, 1998). The extension study at 4 years confirmed the benefit on relapse rate for both doses with a dose-dependent response trend of borderline statistical significance, showing also an effect on time to sustained disability progression in the high dose group and a highly significant reduction in the number of new MRI lesions with a dose dependent effect (PRISMS, 2001). Effect on new T2 lesions was probably similar to that observed with IFNB-1b, but the results were not directly comparable because different measures were reported.
More recent studies comparing different IFNBs efficacy in relation to dose, frequency and routes of administration, suggest a dose/frequency-effect (Durelli, 2002, Panitch, 2002). However, the latter study was aimed to IFNB-1a s.c. approval in U.S. and the follow-up was limited to 48 weeks. In addition a study comparing IFNB-1a 30 mcg i.m., against 60 mcg i.m. weekly did not find differences in clinical and MRI outcomes at 3 years (Clanet, 2002). Finally, post-marketing studies conducted in Italy and Spain on MS treatment in clinical practice did not find significant differences in reducing relapses frequency between the various IFNB formulations (Milanese, Troiano, Montalban, 2005).
In conclusion, these trials showed an effect of IFNB in reducing the frequency of clinical relapses ranging from 18 to 32% against placebo at 2 years. This partial clinical efficacy was strongly corroborated by the much more relevant effects on brain MRI lesions. Although the results on disability, the most important end point for MS patients, have been thus far unconsistent, IFNBs are worldwide currently considered the first line approved treatment for MS.
To our knowledge, no data on possible increased risk of cancer from immunomodulatory drugs have been published.
7.3. Azathioprine in MS.
Azathioprine is an immunosuppressive drug, successfully used since more than thirty years in the treatment of transplant rejection and of several autoimmune reumathologic and neurologic diseases. Published data about its efficacy in MS derived from several trials, most of them involving small numbers of patients and with different clinical course of disease. These trials had been conducted in pre-IFNB years when disease forms were not yet well defined and therefore RCT could not include patients omogeneous for disease form, and when MRI was not widely available. Nonetheless, these studies were suggestive of a partial benefit in clinical outcomes. A meta-analysis of seven RCTs, including the largest on 354 patients of the British and Dutch MS Azathioprine Trial Group (1988) and the preliminary data of an italian trial (Milanese, 1993), analyzed 793 patients and showed a significantly increased probability of freedom from relapses in azathioprine treated (dose 2.5-3 mg/kg daily) against placebo at 3 years, with a slight, borderline benefit also on EDSS (Yudkin, 1991). No further large RCT on azathioprine efficacy in MS was planned in IFNB years, and no data on azathioprine efficacy on MRI are currently available, except a retrospective MRI study on 44 RRMS patients showing a positive effect of azathioprine in reducing the increase of lesional burden (Cavazzuti, 1997).
Concern about the risk of cancer due to this treatment has been raised. In a case-control study involving 23 cancer cases and 69 controls selected out 1191 MS patients, including 2 patients receiving the drug for 1 month or less, the risk was not significantly increased in those treated with azathioprine (OR 1.7, 95% confidence intervals 0.6-4.6); a possible risk increase was suggested only by treatment duration >10 years or 600 grams cumulative dose (Confavreux, 1996). However, recent mortality data of patients enrolled in the British and Dutch Trial did not find significant differences in risk of cancer, from a 3-year course of azathioprine (3.4%, 95% confidence intervals -2.1 to 9.0%) nor in overall mortality (Taylor, 2004). No increase in the relative risk of cancer was found in a study on 201 azathioprine treated MS patients compared to 247 controls (Amato, 1993).
7.4. Rationale.
Efficacy of IFNBs in RRMS is a worldwide accepted issue. Accordingly, in western countries IFNB is approved and reimbursed by most drug regulatory agencies. Recent reviews suggest that azathioprine seems to be as effective as IFNB on clinical and MRI outcomes. The drug, approved and used in Europe for MS treatment (Hommes, 2004), is currently not approved in Italy specifically for this indication. A post-marketing review comparing the probability to be free from relapses at 2 years in MS patients treated with IFNB, glatiramer acetate (GA), or intravenous immunoglobulins with that reported for azathioprine in Yudkin meta-analysis (1991), concluded for equivalent efficacy (Palace, 1997). A more recent analysis of MS treatments used in UK concluded that IFNB, GA and azathioprine produce a similar reduction in the risk of relapses (15-30%) at 2 years and that a head to head comparison between these medications is worth to be tried (Sudlow, 2003). A a systematic review of clinical and cost effectiveness of immunomodulatory drugs in MS suggested that any benefit from IFNB and GA was achieved at very high cost even in terms of adverse eventa (Clegg, 2001), while a Cochrane review showed that the benefit of these treatments on relapses and disability progression was only marginal (Filippini, 2003).
In the present study azathioprine and IFNB efficacy on clinical and MRI endpoints will be compared in a number of MS patients large enough to draw a conclusive evaluation applicable in every day clinical setting and therefore useful both for patients with MS and for neurologists. The lowest cost of azathioprine (less than 500 euro/yr per patient) compared with IFNB (10,000 euro/yr per patient) would be advantageous in terms of cost for avoided event. If this study will confirm the expected results of a non inferiority of azathioprine compared to IFNBs, the current clinical practice in MS could be modified and the available therapeutic alternative could determine a reduction in IFNB prescriptions, with a saving for the National Health System (NHS) of about 60-70 millions euro/yr.
7.5. Preliminary data.
A pilot study conducted by one of the proponents of this project in 32 RRMS patients treated with IFNB-1b or azathioprine found no significant differences between the two treatments in terms of relapses and EDSS, and suggested an improvement in the quality of life in those treated with azathioprine (Milanese, 2001). In addition a recent prospective study of azathioprine efficay on new brain inflammatory lesions in 14 RRMS patients, showed for the first time a significant effect of azathioprine on this outcome measure (Massacesi, 2005), similar to that described for IFNBs under the same experimental conditions.

8. Objectives of the study

In the present study, azathioprine and IFNB relative efficacy on clinical and MRI endpoints will be evaluated in a number of MS patients large enough to draw a conclusive evaluation applicable in every day clinical setting.
This goal will be pursued through a multicenter, randomized, single-masked, controlled study aimed to show the non-inferiority of azathioprine efficacy compared to IFNB in patients with RRMS, under the experimental conditions used in the RCT that allowed approval of IFNBs. The following outcome measures have been selected accordingly:
Primary Efficacy Measure.
• Clinical activity of disease, measured as number of relapses per patients over the two study years.

Secondary Efficacy Measures.
• Changes on EDSS scale at 2 years.
• Brain MRI activity measured as (a) new brain lesion number and volume within 2 years (b) change of brain lesion load.
• Safety profile of the two treatments in terms of frequency and severity of adverse events
• impact on quality of life.
The study is not aimed to evaluate differences among the IFNBs formulations. According to a pragmatic approach, azathioprine will be instead compared with all types of IFNB formulations as a whole group.
If this study will confirm the hypothesized non-inferiority of azathioprine with respects to IFNBs, the current clinical practice in MS will be modified, with advantage for MS cases resistant or intolerant to IFNBs, who could benefit from a therapeutic option already available. A further advantage would be a reduction in IFNB prescriptions resulting in reduced costs for the Italian National Health Service (INHS).

9. Study design

9.1. STUDY POPULATION: MS outpatients from 35 centers of 19 Italians Regions authorised to IFNB prescription by the INHS.
INCLUSION CRITERIA: a) age 18 to 55 years, b) diagnosis of MS (McDonald criteria, 2001) with relapsing-remitting course, c) at least 2 clinical exacerbations in the preceding 2 years, d) baseline EDSS score of 1.0 to 5.5, e) no clinical relapses within 30 days of study entry, f) effective method of contraception if women with childbearing potential, g) signed informed patient consent.
ESCLUSION CRITERIA: a) immunomodulatory or immunosuppressive treatments in the preceding 12 months, b) steroid therapy in the last 30 days before study entry, c) concomitant diseases precluding IFNB or azathioprine treatment, d) pregnancy or breastfeeding, e)inability to give informed consent, f) pathological conditions interfering with MS evolution, g) allergy to paracetamol or known intolerance to azathioprine or IFNB.
9.2.INTERVENTION: Patients will be randomized to two treatment groups. AZATHIOPRINE GROUP: target dose of 3 mg/kg/daily per os divided in 2 or 3 daily administrations will be reached in 4 weeks, starting with 50 mg daily, and individually adjusted according to lymphocyte count that will be carefully monitored. IFNB GROUP: any of the formulations authorized by the INHS and used in clinical practice: IFNB-1b 250 μg s.c. on alternate days Betaferon®), IFNB-1a 30 μg i.m weekly (Avonex®), IFNB-1a 22 μg or 44 μg s.c. thrice weekly (Rebif®). At each centre the IFNB formulation will be assigned by the local treating neurologist. The target dose will be gradually reached in order to avoid or lessen side-effects over 4 weeks (25% of the dose for 2 weeks and 50% for the following 2 weeks, full dose thereafter). The study drugs will be self-administered by the patients after training. Treatment will last two years. Paracetamol will be allowed for IFNB-related side-effects.
This is a single-masked study. At each partecipating centre the patient, aware of treatment allocation, will be assessed by two physicians: 1) the treating neurologist, unblinded to treatment assignment, responsible for the overall medical management of patients, including patient training to self-administration of drugs, monitoring and treating side-effects, checking periodically blood tests results, confirming and treating relapses and managing any event related to the study drug, including its discontinuation; 2) the examiner neurologist, blinded to treatment assignment, will perform neurological examination and EDSS scoring at baseline, follow-up visits, and in case of relapse. Any possible measure will be adopted to ensure his masking at each visit.
9.3. PRIMARY OUTCOME: the cumulative relapse count per patient over 2 years. Relapse is defined as the appearance of new symptoms or the worsening of old ones attributable to MS, accompanied by new objective neurologic signs (change in at least 1 Functional System or EDSS increase of at least 0.5 point) lasting at least 48 hours in absence of fever, provided that at least 30 days are elapsed from the onset of previous relapse (McDonald, 2001). A visit by the treating and examiner neurologist for confirmation of relapse and assessment of its severity within 1 week from relapse onset is required.
SECONDARY CLINICAL OUTCOMES: a) percentages of patients with 0, 1, 2 or more relapses over the course of the study, b) time to first and second relapse, c) relapse severity measured by the maximum EDSS increase during relapse x relapse duration (days), d) number of treatment failures, defined as the number of patients with more relapses during the study than in the preceding 2 years, or with equal relapses number and increase of at least 1 point on EDSS confirmed at 6 months, or occurrence of CTC grade 3 toxicity, or shift to Secondary Progressive course, e) change in EDSS at 2 years, g) quality of life measured on the self-administered MSQOL-54 scale at 1 and 2 years, h) frequency and severity of side-effects assessed every 3 months (expected adverse events and management procedures are reported in APPENDIX I).
SECONDARY MRI OUTCOMES: 1) at 2 years: number of new brain lesions in T2-weighted scans, new brain lesion volume in T2-weighted scans, brain lesion load (volume) in T2-weighted scans, blake hole number and volume in T1-weighted scans, atrophy, 2) at 1 and 2 years: number of brain contrast enhancing lesions in T1-weighted scans.
9.4. RANDOMISATION PROCEDURE: all patients fulfiling the inclusion criteria who siged written informed consent will be randomised by 2 working days (central telephone randomisation). Randomisation will be performed according to a permuted block design, and stratified by EDSS score (≤ 3.5 or >3.5). The randomisation schedule will be obtained using a pc-based pseudo-random number generator. Patient details will be recorded on a randomisation form and sent to the Randomisation Unit by facsimile.
9.5. INFORMATION RETRIEVAL: all eligible patients who will agree to partecipate to the study by signing the written informed consent (Appendix A) will undergo a SCREENING VISIT (see form Appendix B) by the local treating neurologist including history of the disease, evaluation of inclusion and exclusion criteria, neurological examination, previous treatments, concomitant diseases, blood tests, ECG, chest Rx (performed within the preceding 6 months), PAP-test and β-HCG in women, brain MRI. After retrieval of all screening data the patients will be randomized. A BASELINE VISIT will be performed just before starting treatment by the treating and the examiner neurologist (forms in Appendix B). At baseline MSQOL-54 will be self-administered (see flow-chart C).
Blood tests will be monitored every 3 months in all patients and at 2-4-8-12 weeks in Azathioprine treated patients to adjust the dose. A FOLLOW-UP VISIT will be performed every 3 months by the treating neurologist including clinical history updating, blood tests evaluation, concomitant treatments recording, side-effects registration and every 6 months by the examiner neurologist (EDSS, Appendix B,H,I). In case of relapse a RELAPSE VISIT will be performed within 7 days from onset by the examiner neurologist to confirm it and by the treating neurologist for its medical management (forms in Appendix B). All data forms (except serious adverse event forms) will be batched and sent every 6 months from each centre to the trial coordinator. The two remaining CRF copies will be kept on file by the local investigator for possible external inspections.
9.6. CRITERIA FOR STUDY DISCONTINUATION. The study will be discontinuated at any time for safety (incidence of major events i.e. deaths, serious adverse events) and in the case the interim analysis will show a highly significant difference in the primary

endpoint between the two study groups in favour of one study group ($p < 0.01$). Any of the following events will determine treatment withdrawal of the single patients: 1) adverse event considered not tolerable by the patient or his physician, 2) consent withdrawn, 3) occurrence of pregnancy, 4) insufficient compliance to the treatment or protocol violation, 5) failure treatment (see 9.3).
9.7. BRAIN MRI SCANS: will be acquired at baseline, at month 12 and 24, ± 30 days. The images will be acquired as follows:
- magnet power 1 T or more; -slice thickness, 4 mm; "inter-slice gap", none; orientation of the section, axial; matrix, 256 x 256; FOV, 25.6 cm; 2 excitations; sequences for images without medium contrast: double echo T2-weighted,; fFLAIR sequences for images with contrast medium, T1- weighted. Acquisition, 8 min. or more after contrast medium (gadolinium (Gd) i.v., 0.1 mmol/kg). ACQUISITION PROTOCOL: 1) survey T1w; 2) Dp and T2 w; 5) contrast medium administration; 6) fFLAIR; 7) T1 spin echo. Time consume, less than 30 minutes. Bicommessural axial section will be used for precise repositioning. All images of each patient will be acquired with the same parameters standardized along the study. Standardized Brain MRI protocol and schedule for routine MS patient follow up, have been selected for this study. This will allow costs to be covered by the Italian NHS.
MRI DATA EVALUATION. The images will be independently evaluated by two trained neurologists blind to treatment. Number and volume of lesions will be evaluated with semiautomated conturing techniques through dedicated software packages. Definition of the MRI outcome measures: -NEW BRAIN T2 LESIONS: hyperintensities in T2w scans not present at baseline image. Months 12 and month 24 images will be compared with baseline image. -CONTRAST ENHANCING LESIONS: hyperintense lesions when observed in T1w scans after Gd i.v. administration. -TOTAL LESION LOAD: total volume of abnormal hyperintensity in T2w images. -BLACK HOLES: non contrast enhancing ipointensities in T1w scans acquired before Gd administration.
9.8. MONITORING OF THE STUDY: Monitors trained by the Coordinating Centre will visit all centres before start of enrolment and every 4 months thereafter to check organization, verify CFR accuracy and protocol adherence.
9.9. SAMPLE SIZE ESTIMATE: The primary objective is to show the non-inferiority of azathioprine against IFNB in the intention-to-treat population. The a priori equivalence margin M is based on the observed effect of IFNB vs placebo(E-IFNB)on the mean reduction of 0.82 (95% CI, -0.42 to -1.22) relapses over two years, in RRMS under the same experimental conditions of this study (PRISMS,1998; IFNB, 1993).The M value has been selected at a 49% of E-IFNB: 0.82x0.49= 0.40, corresponding to our estimate of the smallest clinically acceptable difference between IFNB vs placebo (as the lower 95% CI of the effect). Azathioprine will be judged non-inferior to IFNB if the upper limit of the one-sided 95% CI of the mean effect of azathioprine vs IFNB (i.e. the difference in the mean relapse number over two years between the two groups), will be lower than 0.40. With a power of 0.8, α of 0.05, taking into account an estimated 20% loss to follow-up, a total sample of 360 patients(175/treatment arm) will be needed. Based on identical considerations, a total of 96 patients per treatment arm (total of 192 patients)will be included in the MRI study for new brain lesion evaluation.
9.10. ORGANIZATIONAL CHARACTERISTICS.
PROPONENT CENTRE: the Department of Neurological and Vision Sciences, University of Verona.
CLINICAL TRIAL COORDINATION: the C. Besta National Neurologic Institute, Milan
MRI COORDINATION: the Department of Neurological and Psychiatric Sciences, University of Florence.
RANDOMISATION CENTER: Neuroepidemiology Unit of the C. Besta National Neurologic Institute.
SAFETY MONITORING: the Department of Farmacology, University of Ancona. INDEPENDENT DATA SAFETY MANAGEMENT COMMITTEE (IDSMC) has been established to oversee the progress of the trial, ensuring that it is conducted and reported in accordance with the protocol, good clinical research practice, and the applicable regulatory requirements, and to monitor the safety data and the critical efficacy endpoints towards its interim and overall objectives.
STEERING COMMITTEE (SC): executive body including the neurologists and neuroepidemiologists who promoted and devised the study. It will: prepare all documents for the study, assure protocol adherence from partecipant centres and respect of timing schedule, organize periodic meetings among investigators, check information from clinical monitors, decide corrective measures when needed, provide for funds distribution, data interpretation, manuscript preparation and data publication. The members of committee structures of the trial and the list of partecipating Centres are reported in Appendix G.
FUND ADMINISTRATION. The Italian Foundation of Multiple sclerosis (F.I.S.M.) will be the carrier of the funds. The Italian Association of Neuroepidemiology will support this study.
9.11. FEASIBILITY: each centre will recruit 8-14 patients and assure a appropriate setting for the study. The coordinators of the study declare that the Institutions that they are afiliated to and those who are involved in the study, have accepted to conduct the present study and agree that the human and technological resources described in the present study protocol will be employed as described.
9.12. TIMING. Study duration: 36 months. Recruitment period: month 0 to 12th. Treatment period: 24 month/patient (months 0 to 36). Interim analysis completed: month 26rd. Final analysis completed: month 38th. Final report provided: month 42th.
9.13. STATISTICAL ANALYSIS. All analyses will be by intention-to-treat. Efficacy analysis will be also carried out after excluding non-compliers (patients who will take less than 80% of therapy) and subjects who drop-out for any reason. Summary statistics of all relevant variables will be tabulated by treatment arm and time (baseline, 12 and 24 months). Baseline data will be compared using t-test, Wilcoxon rank-sum test, c2 test, or Fisher's exact test. Effect of azathioprine on IFNBs for the non-inferiority estimate, will be calculated from the differences and their C.I.s, of the mean relapse number per treatment arm. Relapse count will be compared by Poisson regression model, including as covariates relapse number in the 2 years prior to enrolment, age, baseline EDSS score, and disease duration. Risk of treatment failure will be compared with the log-rank test and multivariate risk determined by proportional-hazards regression model, including the above mentioned covariates. Probability of being relapse-free at 1 and 2 years and risk of one point confirmed EDSS worsening will be compared with the log-rank test and determined multivariately by proportional-hazards regression model. Kaplan-Meier curves will be plotted. MSQOL-54 scale scores changes at 1 and 2 years will be compared with the Wilcoxon rank-sum test. Number of adverse events will be compared by Poisson regression model. All data will be analysed using Stata 8 software (Stata Corporation, College Station, Texas).
9.14. ETHICAL ASPECTS. The study will be performed according to the principles stated in the Declaration of Helsinki and in the Good Clinical Practice Guidelines (European Union, Directive 2001/20/EC).Prior to trial initiation, the study protocol and any other requested documents will be submitted to the Ethics Committee of each centre. Each patient will receive full and adequate information about the study before enrolment, being free to discontinue partecipation at any time. The investigator will ensure patient's anonimity and accuracy of data entered in the CRF. Patients will be identified by number and initials and all data will be stored and analyzed according to national data legislation.

10. References

1. IFNB MS Study Group. Interferon beta-1b is effective in relapsing-remitting multiple sclerosis. I. Clinical results of a multicenter, randomized, double-blind, placebo-controlled trial. Neurology 1993; 43:655-661
2. Kurtzke JF. Rating neurologic impairment in multiple sclerosis: An expanded disability status scale (EDSS). Neurology 1983;33:1444-1452
3. Paty DW, Li DK. Interferon beta-1b is effective in relapsing-remitting multiple sclerosis. II. MRI analysis results of a multicenter, randomized, double-blind, placebo-controlled trial. Neurology 1993; 43:662-667
4. IFNB MS Study Group. Interferon beta-1b in the treatment of multiple sclerosis. Final outcome of the randomized controlled trial. Neurology 1995; 45:1277-1285
5. Jacobs LD, Cookfair DL, Rudick RA et al. Intramuscular interferon beta-1a for disease progression in relapsing MS. Ann Neurol 1996; 39:285-294
6. PRISMS Study Group. Randomized, double blind, placebo-controlled study of interferon beta-1a in relapsing-remitting multiple sclerosis. Lancet 1998; 352:1498-1504
7. PRISMS Study Group, The University of British Columbia MS/MRI Analysis Group. PRISMS-4: Long-term efficacy of interferon-beta-1a in relapsing MS. Neurology 2001;56:1628-1636
8. Poser CM, Paty DW, Scheinberg L et al. New diagnostic criteria for multiple sclerosis: guidelines for research protocols. Ann Neurol 1983;13-227-231
9. Durelli L, Verdun E, Barbero P et al. Every-other-day interferon beta-1b versus once-weekly interferon beta-1a for MS: results of a 2-year prospective randomized multicentre study (INCOMIN). Lancet 2002;359:1453-1460
10.Clanet M, Radue EW, Kappos I et al. A randomized, double-blind, dose-comparison trial of weekly interferon beta-1a in relapsing MS. Neurology 2002; 59:1507-1517
11.Panitch H, Goodin DS, Francis G et al. Randomized, comparative study of interferon beta-1a treatment regimens in MS: The EVIDENCE Trial. Neurology 2002; 59:1496-1506
12.Milanese C, Trojano M, Montalban X. Il trattamento della SM nella pratica clinica. In: Approfondimenti monotematici sulla sclerosi multipla, Gallarate, 17-18 marzo 2005
13.British and Dutch MS Azathioprine Trial Group. Double-masked trial of azathioprine in multiple sclerosis. Lancet 1988; 2:179-183
14.Yudkin PL, Ellison GW, Ghezzi A et al. Overview of azathioprine treatment in Multiple Sclerosis. Lancet 1991; 338:1051-1055
15.Milanese C, La Mantia L, Salmaggi A et al. A double-blind study on azathioprine efficacy in multiple sclerosis: final report. J Neurol 1993; 240:295-298
16.Cavazzuti M, Merelli E, Tassone G et al. Lesion load quantification in serial MR of early relapsing multiple sclerosis patients in azathioprine treatment. A retrospective study. Eur Neurol 1997; 38:284-290
17.Taylor L, Hughes RAC, McPherson K. The risk of cancer from azathioprine as a treatment of multiple sclerosis. Eur J Neurol 2004; 11:141-142
18.Confavreux C, Saddler P, Grimaud J et al. Risk of cancer from azathioprine therapy in multiple sclerosis: a case-control study. Neurology 1996; 46:1607-1612
19. Amato MP, Pracucci G, Ponziani G et al. Long-term safety of azathioprine therapy in MS. Neurology 1993;43:831-833
20.Hommes OR, Weiner HL. Clinical practice of immunosuppressive treatments in MS: results of a second international questionnaire. J Neurol Sci 2004;223:65-67
21.Milanese C, La Mantia L, Salmaggi A, et al. Azathioprine and interferon beta-1b treatment in relapsing-remitting multiple sclerosis. J Neurol Neurosurg Psychiatry 2001; 70:413-14
22.Massacesi L, Parigi A, Barilaro A et al. Efficacy of azathioprine on Multiple Sclerosis new brain lesions evaluated by MRI. Arch Neurol 2005;62:1843-1847
23.Palace J, Rothwell P. New treatments and azathioprine in Multiple Sclerosis. Lancet 1997; 350:261,
24.Sudlow CL, Counsell CE. Problems with UK government's risk sharing scheme for assessing drugs for MS. BMJ 2003; 326:388-392
25.Clegg A, Bryant J. Immunomodulatory drugs for MS: a systematic review of clinical and cost effectiveness. Expert Opin Pharmacother 2001; 2:623-639
26.Filippini G, Munari L, Incorvaia B et al. Interferon in relapsing-remitting MS. A systematic review. Lancet 2003; 361:545-552
27.Cancer Therapy Evaluation Program, Common Terminology Criteria for Adverse Event, Version 3.0, DCTD, NCI, NIH, DHHS, dicembre 2003
28.McDonald WI, Compston A, Elan G et al. Recommended diagnostic criteria for MS: guidelines from the International Panel on the diagnosis of MS. Ann Neurol 2001;50:121-127
29.Vickrey BG, Hays RD, Harooni R, Myers LW, Ellison GW: A health-related quality of life measure for multiple sclerosis. Qual Life Res 1995; 4:187-206.
30.Solari A, Filippini G, Mendozzi L, et al. Validation of Italian multiple sclerosis quality of life 54 questionnaire. J Neurol Neurosurg Psych 1999;67:158-162
31.Mc Cullagh P, Nelder JA. Generalized linear models. 2nd ed. London: Chapman and Hall, 1989

11. Budget

a. Please specify the overall expected costs for each of the items indicated below and for each year of the project

Items	Expected costs for the 1st year (Euro)	Expected costs for the 2nd year* (Euro)	Expected costs for the 3rd year* (Euro)	Total (Euro)
**Personnel**	*540.000,00*	*517.000,00*	*524.000,00*	1.581.000

**Supplies**	*10.000,00*	*0,00*	*0,00*	10.000
**Services**	*10.000,00*	*52.500,00*	*62.500,00*	125.000
**Travels / Meetings/ Courses**	*32.000,00*	*32.000,00*	*32.000,00*	96.000
**Overhead (max 10% of total)**	*6.000,00*	*6.000,00*	*6.000,00*	18.000
**Total (Euro)**	**598.000**	**607.500**	**624.500**	**1.830.000**

b. Please specify person-months for the entire project

Categories of personnel	Person-months
**Researchers**	*1080*
**Technicians/administration**	*108*
**Total**	**1188**

c. Please specify the cost of the main categories of supplies for the entire project

nº	Categories of supplies	Cost (Euro)
1.	*informatic and electronic consumables*	*10.000,00*
	**Total (Euro)**	**10.000**

d. Please specify the distribution of costs between the coordinating centre and the other centres

	Total Costs (Euro)	%
**Coordinating centre**	*753.000,00*	41,15 %
**Transfers to other centres**	*1.077.000,00*	58,85 %
**Total (Euro)**	**1.830.000**	**100**

e. Please specify the expected costs for the main activities carried out by the coordinating centre, for the entire project

nº	Activities	Carried out within the coordinating centre* (Euro)	Carried out in outsourcing (Euro)	TOTALE
1.	***Coordination***	*274.000*		274.000
2.	***Monitoring***	*147.000*		147.000
3.	***Data collection and analysis***	*178.000*		178.000
4.	*consumables*	*10.000*		10.000
5.	*funds administration*		*18.000*	18.000
6.	*travels*		*96.000*	96.000
7.	*patient insurances*		*30.000*	30.000
	**TOTALE**	**609.000**	**144.000**	**753.000**

f. Please specify any additional* expected cost per patient included in the study (excluding the costs indicated in table E: coordination, monitoring, data analysis, etc.) for the entire project

nº	Items	Expected costs (Euro)
	**Total (Euro)**	**0**

12. Disclosure of interest

No Conflict of interest to declare

Yes, Please specify:

13. Annex

APPENDIX A. INFORMED CONSENT

Azathioprine versus Interferon beta for the treatment of Multiple Sclerosis
Multicenter randomised study

INFORMATION FOR THE PATIENT

What does the study propose?
The study has the purpose of comparing the efficacy of interferon beta and azathioprine for the treatment of multiple sclerosis (MS). Interferon beta is a drug used in the relapse form of multiple sclerosis. The Italian Health Ministry has approved three different types of interferon beta, Betaferon, Avonex and Rebif, as all of them proved to be effective in reducing the relapse frequency and the number of cerebral lesions seen with a magnetic resonance. Betaferon and Rebif are administered three times a week subcutaneously, while Avonex intramuscularly by an injection dose once a week.
Azathioprine is an immunosuppressive drug and has probably been the drug most frequently used in the last years for the treatment of multiple sclerosis and other autoimmune diseases. Various clinical studies' results indicate that also azathioprine is able to reduce the relapse rate and it has been shown recently that it prevents the cerebral lesions shown by magnetic resonance imaging. Azathioprine has not yet received formal approval for its use in treating MS.
We hope that the study will give us the necessary information to allow us to extend the use of azathioprine also in multiple sclerosis, to implement the available resources and to improve patient satisfaction.

Which are the side effects of interferon beta and azathioprine?
The most frequently observed side effects after interferon beta administration have been: flu like symptoms such as systemic fever, muscular and articular pains, headache, fatigue, as well as reaction at the injection site, reversible disorders of white blood cells, and alterations of the hepatic functionality and elevation of the liver function tests. Side effects of azathioprine include nausea, diarrhoea, reversible reduction of white and red blood cells (lymphocytes and neutrophiles) and alterations of the hepatic functionality and elevation of the liver function tests.
The effects of interferons and azathioprine on the foetus have not been sufficiently investigated yet.

By whom is the study organized and financed?
It is a national project with 30 participating hospitals, financed by the Agenzia Italiana del Farmaco (AIFA), an organism of public domain which is supervised by the Italian Health Ministry. The coordinating centers are the Istituto Nazionale Neurologico "Carlo Besta" of Milan and the University of Florence.

How is the study organized?
The type of therapy which will be prescribed to you (azathioprine or one of the three interferons) will be decided by a random method (casual) according to a randomization list prepared by the coordinating center (Neurological Institute " C. Besta”).
If you are assigned to the treatment with interferon, your neurologist will decide with you which of the three interferons will be given.
The study's foreseen duration is 2 years.
Information about your state of health and your exam results will be collected for two years.
The study also suggests the evaluation of your quality of life through a questionnaire which will be administered to you by your doctor.

If I accept to participate what will I have to do?
The study will not interfere in any way with the care and the decisions that you, together with your doctor, will take from time to time.
Before beginning treatment you have to undergo a neurological visit and fill in the quality of life evaluation questionnaire. You will have to undergo blood tests, an electrocardiogram, a chest radiography and a brain magnetic resonance imaging.
During the study you will repeat every three months the clinical controls and the laboratory's exams. You will be visited by an examining neurologist and a doctor responsible for the study who will arrange to record any side effects and modify (or suspend) the

treatment if it becomes necessary. You will fill in the quality of life questionnaire after 6 months, 1 and 2 years, and will repeat the magnetic resonance and the electrocardiogram after 1 and 2 years of therapy.
This neurology center will remain in contact with you for 24 months during which the information about your illness and care that you received will be monitored and recorded in a database.
Obviously, your participation in the study will have to be free and voluntary and you will be able to interrupt your participation whenever you want.

If I do not accept?
The refusal to participate or the retirement from the study will not involve any modification in the care your neurologist will provide you and in any case your neurologist will guarantee you the best possible care.

How will the privacy be ensured? (Italian law 675/96)
Results of your participation at this study will be completely confidential.
The information will be gathered in a way which will ensure the data confidentiality. In particular, your personal data (name, surname etc.), will not be passed on to the study coordinating center; only a card with a code number that will be made impossible to identify you, by name.
All the information on the people included into the study will be assembled, and only statistical results will be presented as scientific publications, and your identity will not be recognizable in any way.

What can I do to participate in the study?
If you want to take part in the study it is necessary to sign the informed consent form that will be proposed to you by your Doctor. Once signed, it will be preserved at the coordinating center.
We invite you to ask any questions that you have to your neurologist, who will be available to give you all the explanations concerning the study and its management.

Local center:

Dr..

Tel: .... Fax: .... e-mail: ....

Informed consent form

Azathioprine versus Interferon Beta for Multiple Sclerosis

(one copy for the patient and one copy for the coordinating center)

According to the law N.675/96 (the so called “Law about Privacy”) that rules your rights about your personal data, we need your written consent to use your personal data for the study we are undertaking.
All the data are strictly confidential and we will use your personal data according to the principles of transparency, legality, and honestly; we will protect your privacy and your rights according to the law mentioned above.
The National Neurological Institute Carlo Besta, via Celoria 11 - Milan, will be responsible for the data collection and use. You can contact the Institute if you believe that your rights have been infringed according to the law previously mentioned.
For this study you will be covered by the standard insurance coverage available at our Institute.

I, ...,
(first name) (last name)

had the opportunity to ask questions to elicit a better understanding of the treatment and procedure, so that I can make an informed decision (article n.13, law 675/96) to proceed or to refuse this particular course of medical intervention, Azathioprine versus Interferon beta.

This study has been proposed to me by Doctor...

Therefore, I agree to participate in this study and I give the permission for the use of my personal data according to the law mentioned above (article n.13, law 675/96).

Date..

Patient's signature................................

Neurologist's signature...........................

Appendix B: case forms

Screening form:

IFNB VERSUS AZATHIOPRINE IN RR MS

SCREENING VISIT

Patient ____ / ____ (c / n) Date of birth ___ /__/ ___ (d / m / y) Sex M F

Center ________________________ Tel. ________________ E-mail ______________

Responsable neurologist ____________/__________ (c / n) Signature __________________

Examiner neurologist ____________/__________ (c / n) Signature __________________

Date of visit _____/____/_____ (d / m / y)

HISTORY OF THE DISEASE

Date (m/y)	Symptoms	FS	Duration (days)	Sequelae YES	Sequelae NO
___/___	____________________	P C BS S BB V M O	______	?	?
___/___	____________________	P C BS S BB V M O	______	?	?
___/___	____________________	P C BS S BB V M O	______	?	?
___/___	____________________	P C BS S BB V M O	______	?	?
___/___	____________________	P C BS S BB V M O	______	?	?
___/___	____________________	P C BS S BB V M O	______	?	?
___/___	____________________	P C BS S BB V M O	______	?	?
___/___	____________________	P C BS S BB V M O	______	?	?
___/___	____________________	P C BS S BB V M O	______	?	?
___/___	____________________	P C BS S BB V M O	______	?	?
___/___	____________________	P C BS S BB V M O	______	?	?
___/___	____________________	P C BS S BB V M O	______	?	?

SCREENING VISIT-NEUROLOGICAL EXAMINATION

	FS score	objective signes
P	______	____
		____
BS	______	____
		____
C	______	____
		____
S	______	____
		____
BB	______	____
		____
V	______	____
		____
M	______	____
		____
O	______	____
		____

Deambulation
- ? normal
- ? abnormal without aids for meters ________
- ? abnormal with 1 support for meters ________
- ? abnormal with 2 supports for meters ________
- ? impossible

EDSS _________

CLINICAL FEATURES

n° relapses from onset ________ last 2 yrs ________ last 1 yr ________

EDSS today ________ 2 yrs before ________ 1 yr before ________

n° steroid courses from onset ________ 2 yrs before ________ 1 yr before ________

last relapse onset____/____/____ stable from___/____/____
d m y d m y

last steroid course from ____/____/____ to ___/____/____
d m y d m y

PREVIOUS THERAPIES for MS NO ? YES ? *if YES specify*

Drug	from (m/y)	to (m/y)
____________________	_____/_____	_____/_____
____________________	_____/_____	_____/_____
____________________	_____/_____	_____/_____
____________________	_____/_____	_____/_____

EXAMINATIONS according to PROTOCOL

Exam	date (d/m/y)	result N	result P	specify
Brain MRI	____/___/____	?	?	______________________
ECG	____/___/____	?	?	______________________
Chest RX	____/___/____	?	?	______________________
Pap test	____/___/____	?	?	______________________
β HCG	____/___/____	?	?	______________________
Hematological tests	____/___/____	?	?	______________________
				______________________
				______________________
Biochemical tests	____/___/____	?	?	______________________
				______________________
				______________________

CONCOMITANT DISEASES NO ? YES ? *if YES specify*

Disease	from (m/y)	to (m/y)	ongoing	therapy +/-	from (m/y)	to (m/y)	ongoing
____________	___/___	___/___	?	_______	___/___	___/___	?
____________	___/___	___/___	?	_______	___/___	___/___	?
____________	___/___	___/___	?	_______	___/___	___/___	?
____________	___/___	___/___	?	_______	___/___	___/___	?

INCLUSION CRITERIA (all YES)

	YES	NO
1. The patient is affected by definite MS according to Mc Donald criteria	?	?
2.The patient's age is between 18 and 55 years included	?	?
3.The patient's disease course is of the Relapsing-Remitting type	?	?

4. The patient has had 2 or more relapses in the last 2 years ? ?

5. The patient's disability is between 1 and 5.5 EDSS score included ? ?

6. The patient, being a woman at risk of pregnancy, agrees to adopt an effective contraception during study ? ?

7. The patient is able to understand the objectives and the modalities of the study and to give his consent ? ?

8. The patient, informed of the expected benefits and side effects of IFNB and Azathioprine, and of the study protocol procedures, agrees to participate to the study and has signed a written informed consent ? ?

EXCLUSION CRITERIA (all YES)

1. The patient satisfy all the inclusion criteria ? ?

2. The patient didn't take any Immunomodulatory or Immunosuppressive treatment in the last 12 months ? ?

3. The patient didn't present any relapse of MS in the last 30 days ? ?

4. The patient didn't take steroids in the last 30 days ? ?

5. The patient is not affected by any disease which could be worsened by IFNB and Azathioprine, or could interfere with IFNB or Azathioprine treatment ? ?

6. The patient is a woman and is not pregnant and is not breast-feeding ? ?

7. The patient is a male, or is a woman not at risk of pregnancy ? ?

8. The patient do not present any known intollerance either to paracetamolo and FANS, and to IFNB and Azathioprine ? ?

Baseline form (treating neurologist)

IFNB VERSUS AZATIOPRINA NELLA SMRR

BASELINE VISIT

Iniziali Paziente ____/____ Randomizzazione Farmaco __________ Codice ___/___/___
c n

Centro ______________________ Medico *responsabile* ____________/____________
c n

Data compilazione____/____/____ Inizio trattamento ____/____/____
g m a g m a

Dalla visita di screening il paziente

ha presentato NUOVE RICADUTE ? NO ? SI ? se SI specificare

sintomo/i	esordio (g/m/a)	fine (g/m/a)	in corso
______________________	___/___/___	___/___/___	?
______________________	___/___/___	___/___/___	?
______________________	___/___/___	___/___/___	?

ha effettuato TERAPIA STEROIDEA? NO ? SI ? se SI specificare

farmaco	inizio (g/m/a)	fine (g/m/a)	in corso
______________________	___/___/___	___/___/___	?
______________________	___/___/___	___/___/___	?
______________________	___/___/___	___/___/___	?

ha presentato ALTRE PATOLOGIE? NO ? SI ? se SI specificare

malattia	esordio (g/m/a)	fine (g/m/a)	in corso
______________________	___/___/___	___/___/___	?
______________________	___/___/___	___/___/___	?
______________________	___/___/___	___/___/___	?

conferma il CONSENSO a partecipare allo studio? NO ? SI ? se NO specificare

motivo/i ______________________________________
____
____

Baseline form (examiner neurologist)

BASELINE VISIT

Iniziali Paziente ____/____ (c / n) Randomizzazione codice ___/___/___

Centro ________________________ Medico *esaminatore* _______________/_____________ (c / n)

Data compilazione ____/____/____ (g / m / a) Inizio trattamento ____/____/____ (g / m / a)

ESAME NEUROLOGICO

	FS score	descrizione
P	_______	____
TE	_______	____
C	_______	____
S	_______	____
Sf	_______	____
V	_______	____
M	_______	____
A	_______	____

Deambulazione
? normale
? patologica senza appoggio per metri ________
? patologica con 1 appoggio per metri ________
? patologica con 2 appoggi per metri ________
? impossibile

EDSS _________

Follow-up form

4.The patient has had 2 or more relapses in the last 2 years ? ?

5.The patient's disability is between 1 and 5.5 EDSS score included ? ?

6.The patient, being a woman at risk of pregnancy, agrees to adopt an effective contraception during study ? ?

7.The patient is able to understand the objectives and the modalities of the study and to give his consent ? ?

8.The patient, informed of the expected benefits and side effects of IFNB and Azathioprine, and of the study protocol procedures, agrees to participate to the study and has signed a written informed consent ? ?

EXCLUSION CRITERIA (all YES)

1.The patient satisfy all the inclusion criteria ? ?

2.The patient didn't take any Immunomodulatory or Immunosuppressive treatment in the last 12 months ? ?

3.The patient didn't present any relapse of MS in the last 30 days ? ?

4.The patient didn't take steroids in the last 30 days ? ?

5.The patient is not affected by any disease which could be worsened by IFNB and Azathioprine, or could interfere with IFNB or Azathioprine treatment ? ?

6.The patient is a woman and is not pregnant and is not breast-feeding ? ?

7.The patient is a male, or is a woman not at risk of pregnancy ? ?

8.The patient do not present any known intollerance either to paracetamolo and FANS, and to IFNB and Azathioprine ? ?

FOLLOW UP VISIT

Patient ____ / ____ Randomization Drug ___________Code ___/___/___
c n

Center _________________________ ***Responsible*** neurologist______________/______________
c n

Date of visit____/____/____ months from entry _3_/ 6 /_9 /_12_/_15_/_18_/_21_/_24_/
d m y

During the last 3 months the patient

has presented NEW RELAPSES? NO ? YES ? *if YES specify*

onset (d/m/y)	stable from (d/m/y)	ongoing	Mx EDSS	final EDSS
__/__/__	__/__/__	?	_________	_________
__/__/__	__/__/__	?	_________	_________

has suffered SIDE EFFECTS? NO ? YES ? *if YES specify*

side effect *	onset (d/m/y)	duration (days)	ongoing	Hospital +/-	recovery **
_____________	__/__/__	______	?	______	______
_____________	__/__/__	______	?	______	______
_____________	__/__/__	______	?	______	______
_____________	__/__/__	______	?	______	______

has presented OTHERS DISEASES? NO ? YES ? *if YES specify*

disease	onset (d/m/y)	duration (days)	ongoing	Hospital +/-	recovery **
_____________	__/__/__	______	?	______	______
_____________	__/__/__	______	?	______	______

has taken OTHERS THERAPIES? NO ? YES ? *if YES specify*

drug	from (d/m/y)	to (d/m/y)	ongoing	reasons
_____________	__/__/__	__/__/__	?	________________________
_____________	__/__/__	__/__/__	?	________________________
_____________	__/__/__	__/__/__	?	________________________

has been submitted to OTHERS EXAMINATIONS? NO ? YES ? *if YES specify*

exam	date (d/m/y)	normal	abnormal	specify
_____________	__/__/__	?	?	________________________
_____________	__/__/__	?	?	________________________

Continues treatment at protocol dose ? from __/__/__ (d m y) *if not specify*

at decreased dose ? at increased dose ? dose ________________ from __/__/__ (d m y)

Discontinues treatment definitively ? from __/__/__ (d m y) *if not specify*

temporary discontinuation ? from __/__/__ (d m y) to __/__/__ (d m y)

Reasons for treatment discontinuation

side effects ? pregnancy planned ? ongoing ?

increased n° relapses ? no consent ?

EDSS increase ? 1 ? other disease ? ______________________ (specify)

shift to SP course ? death ? ______________________ (reason)

protocol violations ? lost to follow up ? from __/__/__ (d m y)

OTHERS THERAPIES prescriptions NO ? YES ? *if YES specify*

drug	from (d/m/y)	to (d/m/y)	reason
____________	__/__/__	__/__/__	____________
____________	__/__/__	__/__/__	____________
____________	__/__/__	__/__/__	____________
____________	__/__/__	__/__/__	____________
____________	__/__/__	__/__/__	____________

OTHERS EXAMINATIONS prescriptions NO ? YES ? *if YES specify*

exam	date (d/m/y)	reason
____________	__/__/__	____________
____________	__/__/__	____________
____________	__/__/__	____________
____________	__/__/__	____________

FOLLOW UP VISIT

Patient ____/ ____ (c / n)   Randomization code ___/___/___

Center _________________________   ***Examiner*** neurologist ________________/_____________ (c / n)

Date of visit ____/____/____ (d / m / y)   months from entry _3_/ 6 /_9 /_12_/_15_/_18_/_21_/_24_/

NEUROLOGICAL EXAMINATION

	FS score	objective signes
P	_______	______________________________
BS	_______	______________________________
C	_______	______________________________
S	_______	______________________________
BB	_______	______________________________
V	_______	______________________________
M	_______	______________________________
O	_______	______________________________

Deambulation
- ? normal
- ? abnormal without aids for meters ________
- ? abnormal with 1 support for meters ________
- ? abnormal with 2 supports for meters ________
- ? impossible

EDSS

Relapse form (treating neurologist)

- D - RELAPSE VISIT

Patient ____ / ____ (c / n)   Randomization   Drug __________ Code ___/___/___

Center ______________________   ***Responsable*** neurologist ____________/____________ (c / n)

Date of I° visit ____/____/____ (d m y)   (within 7 days from onset)

from the last visit of ____/____/____ (d m y) the patient has presented

new neurological symptoms ?   worsening of previous symptoms ?   *specify*

symptom	onset (d/m/y)	fever +/-	evolution ***
____________________________	___/___/___	______	__________

I° N.E. performed by *examiner* neurologist ____/____/____ (d m y)

F.S. worsened by ? 1   P C BS S BB V M O

EDSS today _______   pre relapse _______   EDSS change _______

Relapse confirmed   YES ?   NO ?   *if YES*
Steroid treatment   YES ?   NO ?   *if YES specify*

drug	dose/day/i.m.-i.v.-os	from (d/m/y)	to (d/m/y)
________________	_______________	___/___/___	___/___/___

Date of II° visit ____/____/____ (d m y)   (at 4 +/-1 weeks from onset)

II° N.E. performed by *examiner* neurologist ____/____/____ (d m y)

Remission without sequelae ?   with sequelae ?   ongoing ?

in case of remission, with or without sequelae, specify

relapse duration _________ days
EDSS today _________   pre relapse _______   EDSS change_______

if still ongoing, plan a next visit at 8+/-1 weeks from onset ____/____/____ (d m y)

CRF Legenda (see Follow up visit and Relapse visit)

** Side Effects*
1) Flou like syndrome, 2) Fever, 3) Injection site reaction (eritema +/- edema), 4) injection site cutaneus necrosis, 5) Systemic allergic reaction, 6) nausea-vomiting, 7) Others (specify), 8) Abnormal hematological tests (8.1 decreased WBC count, 8.2 decreased Lynphocite count, 8.3 decreased RBC count, 8.4 decreased Hb, 8.5 increased G.V., 8.6 decreased Patelets, 8.7 others), 9) Abnormal biochemical tests (9.1 increased ESR, 9.2 increased ALT, 9.3 increased AST, 9.4 increased GGT, 9.5 abnormal tiroid function, 9.6 increased antitiroid Abs).
Note: 1), 2), 3) still present in the previous 3 months, must be considered ongoing

8), 9) duration is the interval between the abnormal tests and the subsequent normal one; if the subsequent test is still abnormal or not performed, is ongoing.

*** Recovery*
1) Remission without sequelae, 2) Remission with mild sequelae (not interfering with daily life nor with protocol treatment), 3) Remission with severe sequelae (interfering with daily life or requiring adjustement or temporary discontinuation of protocol treatment).

**** Evolution of relapse symptoms from onset to first visit*
1) Stable, 2) worsened, 3) Improved.

Appendix C: flow-chart

FLOW-CHART OF THE STUDY

	Screening	0 Baseline	1 month	3 month	6 month	9 month	12 month	15 month	18 month	21 month	24 month
**Informed Consent**	x										
**Inclusion form**	x										
**Neurologic assessment, EDSS**	x	x		x	x		x		x		x
**MSQOL-54§**		x			x		x				x
**ECG**	x										
**Chest Rx (within the preceding 6 months)**	x										
**Erytrocytes, hemoglobin, leukocytes with differential count, platelets, ALT, AST, GGT, ALP, bilirubin °**	x		x	x	x	x	x	x	x	x	x
**ANA, TSH, FT3, FT4, anti-thyroid ab °°**	x				x		x		x		x
**PAP-test and β-HCG in women**	x										
**TPMT test ^**		x									
**IFNB NA test ^^**							x				x
**Follow-up form***			x	x	x	x	x	x	x	x	x
**Relapse form ****											
**Adverse events form**			x	x	x	x	x	x	x	x	x
**Brain MRI**	x						x				x

§ self-administered
° the azathioprine group will have these hemato-chemical tests every 2 weeks for the first month, then every 30 days for the second and third mont then every 3 months
°° the azathioprine group will have these tests only at baseline, the IFNB group every 6 months
^ tiopurine metyltransferase test (only in azathioprine group)
^^ IFNB neutralizing antibodies test (only in IFNB group)
* including concomitant treatments
** at any relapse

EXPANDED DISABILITY STATUS SCALE (EDSS)

*0.0 = Normal neurological exam (all grade 0 in FS *)*

*1.0 = No disability , minimal signs in one FS * (i.e. grade 1)*

*1.5 = No disability , minimal signs in more than one FS * (more than one FS grade 1)*
2.0 = Minimal disability in one FS (one FS grade 2 , others 0 or 1)
2.5 = Minimal disability in two FS (two FS grade 2 , others 0 or 1)
3.0 = Moderate disability in one FS (one FS grade 3, others 0 or 1) or mild disability in three or four FS (three or four FS grade 2, others 0 or 1) though fully ambulatory
3.5 = Fully ambulatory but with moderate disability in one FS (one grade 3) and one or two FS grade 2; or two FS grade 3; or five FS grade 2 (others 0 or 1)
4.0 = Fully ambulatory without aid , self-sufficient , up and about some 12 hours a day despite relatively severe disability consisting of one FS grade 4 (others 0 or 1), or combinations of lesser grades exceeding limits of previous steps; able to walk without aid or rest some 500m
4.5 = Fully ambulatory without aid, up and about much of the day, able to work a full day, may otherwise have some limitation of full activity or require minimal assistance; characterized by relatively severe disability usually consisting of one FS grade 4 (others 0 or 1) or combinations of lesser grades exceeding limits of previous steps; able to walk without aid or rest some 300m.
5.0 = Ambulatory without aid or rest for about 200m; disability severe enough to impair full daily activities (e.g. to work a full day without special provisions); (Usual FS equivalents are one grade 5 alone, others 0 or 1; or combinations of lesser grades usually exceeding specifications for step 4.0)
5.5 = Ambulatory without aid or rest for about 100m; disability severe enough to preclude full daily activities; (Usual FS equivalents are one grade 5 alone, others 0 or 1; or combination of lesser grades usually exceeding those for step 4.0)

6.0 = Intermittent or unilateral constant assistance (cane, crutch , brace) required to walk about 100m with or without resting; (Usual FS equivalent are combinations with more than two FS grades 3+)
6.5 = Constant bilateral assistance (canes, crutches, braces) required to walk about 20 without resting ; (Usual FS equivalents are combinations with more than two FS grades 3 +)
7.0 = Unable to walk beyond approximately five meters even with aid, essentially restricted to wheelchair; wheels self in standard wheelchair and transfers alone; up and about in wheelchair some 12 hours a day; (Usual FS equivalents are combinations with more than one FS grade 4+ ; very rarely pyramidal grade 5 alone)
7.5 = Unable to take more than a few steps; restricted to wheelchair; may need aid in transfer; wheels self but cannot carry on in standard wheelchair a full day; May require motorized wheelchair; (Usual FS equivalents are combinations with more than one FS grade 4 +)
8.0 = Essentially restricted to bed or chair or perambulated in wheelchair, but may be out of bed itself much of the day; retains many self-care functions; generally has effective use of arms; (Usual FS equivalents are combinations generally grade 4+ in several systems)
8.5 = Essentially restricted to bed much of day has some effective use of arm(s); retains some self-care functions; (Usual FS equivalents are combinations generally 4 + in several systems)
9.0 = Helpless bed patient; can communicate and eat; (Usual FS equivalents are combinations, mostly grade 4 +)
9.5 = Totally helpless bed patient; unable to communicate effectively or eat/swallow; (Usual FS equivalents are combinations , almost all grade 4 +)
10.0 = Death due to MS

** Excludes cerebral function grade 1*

GUIDELINES FOR EDSS COMPILATION

NOTE 1:
EDSS steps 1.0 to 4.5 refer to patients who are fully ambulatory , and the precise step number is defined by the Functional Systems (FS) score(s) . EDSS steps 5.0 to 9.5 are defined by the impairment to ambulation and usual equivalents in Functional Systems scores are provided .
NOTE 2 :
EDSS should not change by 1.0 step unless there is a change in the same direction of at least one step in at least one FS . Each step (e.g. 3.0 to 3.5) is still part of the DSS scale equivalent
(i.e. 3). Progression from 3.0 to 3.5 should be equivalent to the DSS score of 3.
NOTE 3 :
Category 1 in the functional systems usually implies that the patient is not aware of the deficit and this deficit does not interfere with normal daily activities .
NOTE 4 :
EDSS grades 6.0 and 6.5 contain both a description of assistance required and walking range .
In general the descriptions given at these EDSS scores are valid .
However the following exceptions are made :
- If a patient is able to walk considerably longer than 100m with 2 sticks , crutches or braces , he is in category 6.0
- If a patient is able to walk more than 10m and less than 100m with 2 sticks , crutches or braces, he is in grade 6.5
- If he needs assistance by another person and is not able to walk more than 50m with 1 stick ,crutch or brace he is in grade 6.5
NOTE 5 :
For calculation of the EDSS grades of the visual function system are introduced as follows : 6 as 4 , 5 and 4 as 3 and 2 as 2 , 1 as 1 .

Appendix G.
ORGANIZATION
Coordinating Centres:
1. CLINICAL COORDINATON. C.Besta National Neurologic Institute, Milan (Clara Milanese, MD, responsible for the MS Centre) and trial coordination (Laboratory of Epidemiology, responsible Graziella Filippini, MD).
2.BRAIN MRI COORDINATION. Department of Neurological and Psychiatric Sciences, University of Florence(Luca Massacesi, MD).

Randomization Unit: Laboratory of Epidemiology, the C.Besta National Neurologic Institute (Alessandra Solari, MD).

IDSMC will be chaired by Gianni Tognoni, pharmacologist and statistician. Other members will be Roberto D'Alessandro, neurologist with expertise in neuroepidemiology, and Leandro Provinciali, neurologist with expertise in Public Health.

Members of SC: Maria Donata Benedetti, MD°, Graziella Filippini, MD, Loredana La Mantia, MD°, Luca Massacesi, MD**, Clara Milanese, MD°, Alessandra Solari, MD*, Gianluigi Mancardi, MD**, Salvatore Amoroso, MD, Mario Battaglia, MD, Gioacchino Tedeschi, MD***

** Members of Statistical subcommittee also (with Valsecchi, MD)*

° Members of Clinical subcommittee also

*** Members of MRI subcommittee also with Carlo Pozzilli, MD*

The Multicenter trial will include:

University of Cagliari (Marrosu)

Aosta Regional Hospital (Bottacchi)
Novara Hospital (Leone)
Molinette Hospital, Torino
San Luigi Hospital, Orbassano (Bertolotto)
University of Genova (Mancardi)
Carlo Besta National Neurologic Institute, Milan (Milanese, La Mantia)
Niguarda Hospital, Milan (Protti)
S.Gerardo Hospital, Monza (Cavaletti)
Clinica Neurologica, University of Verona (Benedetti)
Maggiore Hospital, Verona (Moretto)
Regional Hospital, Bolzano (Schoenhuber)
Santa Chiara Hospital, Trento (Orrico)
MS Centre, San Bortolo Hospital, Vicenza (Bortolon)
University of Bologna (D'Alessandro)
University of Ferrara (Tola)
Ospedale Bufalini di Cesena (Malagù)
Ospedale Morgagni-Pierantoni di Forlì (Neri)
University of Modena (Merelli)
Ospedale Infermi di Rimini (Pasquinelli)
Ospedale S.Maria Nuova di Reggio Emilia (Motti)
Ospedale Ramazzini di Carpi (Santangelo)
University of Florence, Careggi University Hospital (Massacesi)
University of Siena, "Le Scotte" University Hospital (Ulivelli)
Ospedale di Lucca (Mazzoni)
Livorno ASL6 Toscana Hospital (Meucci)
University of Chieti (Lugaresi)
San Camillo Hospital, Rome (Gasperini)
University La Sapienza, Rome (Pozzilli)
University of Sassari (Rosati)
University of Napoli (Cotrufo)
University of Napoli Neurologia II (Tedeschi)
OSpedale di Salerno (Juliano)
University of Palermo (Savettieri)
University of Calabria (Quattrone)

Appendix H.
Expected adverse events
In IFNB treated patients: headache, influenza-like symptoms, injection-side reactions, fatigue, myalgia, fever, nausea, chills; leukopenia, lymphopenia, neutropenia, trombocytopenia, transaminases elevation. For grade 1 AE occurrence only monitoring and no treatment is required, for grade 2 or 3 AE occurrence IFNB will be decreased to half dosage or temporarily discontinued until normality or grade 1, then gradually increased to half dosage or full dose. Treatment will be definitely discontinued in the case of repeated occurrence of grade 3 AE.
In azathioprine treated patients: leukopenia, lymphopenia, neutropenia, trombocytopenia, more than grade 2 of the NCI CTC, transaminases elevation, macrocytosis; rarely: nausea, vomit or anorexia, skin light hypersensibility reactions, alopecia, cough, jaundice

Appendix I.
Criteria for Treatment modification
Following treatment related adverse events onset (grade 2 CTC; grade 3 CTC as for lymphocyte and leucocyte number in azathioprine treated individuals; fever or skin lesion in injection site for IFN patients), the treating neurologist can decrease drug dose or administration frequency according to the severity of the events. As for thrice weekly IFNs, firstly frequency of administration will be reduced; if this will not be enough each dose will be decreased by 50%; as for azathioprine the dose will be reduced starting from 1/2 mg Kg/day at the nearest 25th mg (half tablet).
Criteria for patient withdrawal from the study
The following events will determine treatment withdrawal in individual patients: 1) severe adverse event considered not tolerable by the patient or his physician, (grade 3 CTC; grade 4 CTC as for lymphocyte and leucocyte number in azathioprine treated individuals; 2) consent withdrawn; 3) occurrence of pregnancy, 4) insufficient compliance to the treatment or protocol violation; 5) treatment failure, defined as the occurrence of a relapse number over the course of study higher than in the 2 years preceding study entry, or equal number of relapses with progression of disability of 1 point on EDSS confirmed at 6 months.

NCI Common Toxicity Criteria (CTC)
Severity of adverse experiences and tumor-related symptoms should be graded using the NCI Common Toxicity Criteria as determined by the investigator or reported to him/her by the patient.
For adverse experiences and tumor-related symptoms not included in the common Toxicity Criteria, a grade will be assessed as follows:

Mild = Grade 1
Moderate = Grade 2
Severe = Grade 3
Life Threatening = Grade 4

Ref. of the Proposal

**Last name(family name)**	BENEDETTI	**First name**	MARIA DONATA
**Email**	mariadonata.benedetti@azosp.vr.it	**Tel.**	0458074285
**Fax**	045585933	**Mobile**	

25/01/2006 21:01
