# Supplementary material for: Azathioprine versus Beta Interferons for Relapsing-Remitting Multiple Sclerosis: A Multicentre Randomized Non-Inferiority Trial
Source: PLoS One. 2014 Nov 17;9(11):e113371. doi: 10.1371/journal.pone.0113371 (PMC4234663; doi:10.1371/journal.pone.0113371)
Supplement: Amendment S1 — Amendment to the protocol. (PDF) [file pone.0113371.s003.pdf]

# REVISED STUDY PROTOCOL

## Title

### MULTICENTER RANDOMIZED CONTROLLED STUDY OF AZATHIOPRINE VERSUS INTERFERON BETA IN RELAPSING-REMITTING MULTIPLE SCLEROSIS (M.A.I.N. TRIAL)

## Coordinating Investigators

Clara Milanese MD

Prof. Luca Massacesi

### Steering Committee

Prof. Salvatore Amoroso, Prof. Mario Battaglia, Maria Donata Benedetti, MD, PhD, Graziella Filippini, MD, Loredana La Mantia, MD, Prof. Luigi Mancardi, Daniele Orrico, MD, Prof. Carlo Pozzilli, Alessandra Solari, MD, Prof. Gioacchino Tedeschi.

## Introduction

In the two years from January 2007 to February 2009, MAIN Trial randomized 150 subjects against the 360 planned. To date the Coordinating Center received the CRFs of 139 subjects (see the First Year descriptive analysis, Addendum II).

MAIN Trial Steering Committe after a two-year recruitment decided:

1. to stop the recruitment
2. to continue the study in consideration of its high informative potential despite the relevant change in sample size

The aims of this report are:

1. to justify the stopping of the recruitment after a two year period
2. to prove the study feasibility even with a sample size smaller than expected
3. to give reasons for the request of a formal approval of amendments modifying the study protocol as for the above-mentioned two items.

## End of the recruitment period

The lower than planned recruitment has been determined by the following reasons:

1) only 30 out 50 Centers initially stating their willingness to be involved in the study applied for formal Ethics Committee (ECs) approval

2) the formal approval from all local ECs of participating Centers required a long time (eighteen months since the Coordinating Center EC approval in August 2006 and 14 months since the study onset), the last Center becoming active in April 2008. Therefore, the monthly recruitment rate since the start of the study in February 2007 (Addendum II) increased very slowly reflecting the consecutive activation of Centers which were at the same time obtaining the local EC approval. In the spring 2008 all Centers have started patient recruitment and since then the rate remained steady.

3) the new reimbursement criteria approved in Italy from AIFA in the summer 2008 for IFNB prescription in Multiple Sclerosis (MS), did not require any more the presence of at least 2 relapses in the last two years, thus allowing to start therapy in MS cases since their the first attack. However, MAIN Trial was based on the previous prescription criteria effective in Italy when this trial started (diagnosis of MS according to McDonald criteria, 2001, with at least 2 relapses in the previous two years), essentially identical to those used in the historical RCTs on which MAIN Trial was based. This change in inclusion criteria drastically reduced the number of eligible patients from the participating Centers: the recruitment rate since September 2008 changed over from an average of

10 to 2 subjects/month and was then steady until the end of recruitment (Addendum II), being obviously inadequate to meet in a reasonable time the sample size planned in the study protocol.

Because of all these considerations the Steering Committee decided to stop the recruitment and to evaluate the scientific relevance of the information eventually conveyed by the study.

**Scientific informative potential of the study**

Careful evaluation brought to the conclusion that the study was still informative even with a sample size smaller than estimated and mantained its original scientific relevance.

In fact, **even studying 150 patients, the MAIN Trial still preserves a probability between 60 to 65% to prove the experimental hypothesis on the primary end-point (with p= 0.05), and of 80% on the secondary end-point (with p=0.05).**

This conclusion comes from:

1) redefinition of statistical power and non-inferiority margin referred to the primary end-point and calculated on the basis of new historical data as reported below (see next section)
2) new estimation of final number of drop-outs based on the descriptive analysis of 1rst year (Addendum II, table II); this number should be lower than expected, balancing out at least in part the smaller size of the recruited sample
3) MRI secondary end-point keeping a statistical power up to 80%, since a higher than expected proportion of patients entered the MRI Protocol, with an overall number of recruited cases for this outcome measure close to that planned in the original Protocol
4) high quality of recorded information (see descriptive analysis, Addendum II, table I), so that such data would be useful in meta-analytic studies including previous similar studies

**Informative potential of primary end-point**

New data from a recent Randomized Controlled Trial (RCT) aimed to compare the efficacy of a new treatment (Alemtuzumab) against IFN beta 1a as “active comparator” (Coles et al., NEJM 2008, 359:1786) showed a definite reduction in the number of expected events in the primary end-point and an even greater standard deviations reduction. This result suggested a new computation of MAIN Trial statistical power, regardless of the change in sample size, based on a new estimate of the non-inferiority margin M (μ) and. According to the non-inferiority method guidelines only studies recruiting a patient population qualitatively similar to that of the historical reference studies are considered methodologically sound. As for relapse number, the MAIN Trial actually recruited a

population more similar to that of Coles study than to those included in historical RCT of the 90's used as reference in the original protocol, so that it seems now appropriate to update the MAIN Trial power calculation, the non-inferiority margin μ as well as sample size, according to the observations derived from such recent study.

In fact, the study of Coles et al. is relevant for the purpose of this report because as in MAIN Trial: 1) unlike other recent studies, it defined MS according to Mc Donald criteria, relapsing-remitting form, with at least 2 relapses in previous two years as inclusion criteria; 2) it used the same primary and secondary end-point measures; - 3) the study was conducted after IFNB approval and early prescription use; - 4) the recruitment was done mainly in Europe.

The results of Coles et al. study showed that:

1) the relapse rate/2 yrs observed in IFNB patients (0.72± 0.56) was much lower (= 41%) than the rate observed in the historical RCT arms treated with the same IFNB type and dosage used as reference for MAIN trial (=1.74);

2) relapse frequency variability was proportionally even lower than the relapse number observed in historical RCTs;

3) the mean relapse number in the previous 2 years was lower (77%; = 2.4 relapses, the same observed in MAIN Trial) than in PRISM study (= 3.1 relapses) where the same IFNB type was used.

[Moreover, in three recent RCTs the same change in relapse rate in the arm treated with IFNB was observed, even if data from these studies are less likely to be applicable for the purposes of this report, since the inclusion criteria allowed to recruit also patients with Clinically Isolated Syndrome (CIS, i.e. cases with only one attack). These RCT are: - REGARD: 0.6 relapses in 2 years (no SD reported, *Lancet Neurology* 2008, 7:903); - BECOME: 0.7 relapses in 2 years (no SD reported, *Neurology* 2009, 72:1976); -Fingolimod vs IFN Beta 1a IM: 0.66 relapses/2 years (0.33/yr, 95% CI 0.12-0.21; *NEJM*, e-pub January 2010) .

In two further studies conducted in the same years, Fingolimod vs Placebo, Cladribina vs Placebo the number of relapses in the Placebo treated arm was 0.8/2yrs (0.4/yr, 95% CI 0.34-0.47) and 0.66/2yrs (0.33/yr, 95% CI 0.29-0.38), respectively, while in historical RCTs of the '90s the number of relapses/2 yrs was in mean = 2.5].

Overall, the above-mentioned data support the hypothesis that the populations of patients included in IFNB recent clinical trials have a lower annual relapse rate compared to historical RCTs. This is probably due to the recruitment of patients increasingly less active, and in any case less active of those recruited in historical RCTs. In this respect the MAIN Trial population is similar to that of Coles et al. (Table I, Addendum II), both for disease activity (number of relapses/previous 2 yrs/patient: MAIN trial = 2.40; Coles et al. = 2.42), and disability at entry ( MAIN: EDSS = 2.0; Coles at al. 2008: EDSS = 1.9).

As the non-inferiority margin of the MAIN trial was calculated in term of absolute difference in relapse numbers between placebo and IFN, the lower absolute number of expected events in the MAIN trial based on the study of Coles et al. (2008) implies a new calculation of the theoretic effect $_{\text{IFNbeta - placebo}}$ and its 95% CIs.

Therefore, this new calculation has to be applied to MAIN Trial, in which the recruitment has been conducted under conditions similar to the Cole et al study, i.e. when IFNB, the *comparator* drug, was already largely used in the routine treatment of early MS. Consequently, the non-inferiority margin μ also has to be revised, and in order to maintain *clinical meaningfulness* it must reflect the new expected number of events and standard deviations. However, because of the large variability of these two parameters between the reference studies and the most recent ones, it is difficult to provide a μ estimate in term of absolute number of events, while it is possible in terms of a ratio as well as of a % of the Effect $_{\text{IFNbeta – placebo,}}$ that are quite homogeneous between studies.

**μ estimation as % of the Effect $_{\text{IFNbeta - placebo}}$ and its SD**

The consistent results of the RCTs that in the past established efficacy of IFNB vs. placebo, allow therefore us to estimate the number of expected relapses/2 years (and the annualized relapse rate) in a theoretic placebo arm as 1.45 of the number of events observed in the IFNB arm. Therefore given 0,72 the number of relapses/2 yrs/patient observed in the IFNB arm of the most recent RCT (Coles et al., 2008), the expected number of relapses in a theoretic placebo arm should be 0.72 x 1.45= 1.04. The SD of this hypothetical placebo arm, needed to calculate the CIs of the theoretical Effect $_{\text{IFNbeta - placebo}}$ , can be estimated using the ratio between the SD observed in Coles at al. and the mean value observed in the placebo arm of historical RCTs. As this ratio is 0.73, the estimated DS was 0.80.

Assuming as constant the relapse ratio IFNB arm/theoretic placebo arm, μ can also be estimated not only as a fraction of the difference between the absolute relapse number in the placebo and IFN arms but also as a fraction of the mean absolute relapse number in the IFNB arm. Such a ratio eventually resulted consistently around 0.5 of the number of events observed in the IFNB arm. Since μ was chosen as 50% of such value, it will result equivalent to 25% of N. In the following Table are shown the different values expected according to the different possible values of N:

| Mean number of relapses/2yrs/patient (N) | | Theoretic Effect $_{IFN-Pl}$ | Theoretic Effect $_{IFN-Pl}$ / absolute n. events IFNB | absolute **μ** / absolute n. of events in IFN arm | absolute **μ** |
|---|---|---|---|---|---|
| IFNbeta mean± SD | Placebo mean± SD | | | | |
| 1.74 ± 1.74 | 2.55 ± 1.87 | 0.81 | 0.5 | 25% | 0.41 |
| 1.4± 1.08 | 2.10± 1.53 | 0.7 | 0.5 | 25% | 0.35 |
| 1.3± 1.0 | 1.95± 1.42 | 0.65 | 0.5 | 25% | 0.32 |
| 1.2± 0.93 | 1.8± 1.3 | 0.6 | 0.5 | 25% | 0.3 |
| 1.1± 0.85 | 1.65± 1.2 | 0.55 | 0.5 | 25% | 0.27 |
| 1.0± 0.78 | 1.5± 1.1 | 0.5 | 0.5 | 25% | 0.25 |
| 0.9± 0.70 | 1.35± 0.98 | 0.45 | 0.5 | 25% | 0.225 |
| 0.8± 0.62 | 1.2± 0.88 | 0.4 | 0.5 | 25% | 0.21 |
| 0.72± 0.56 | 1.1± 0.80 | 0.38 | 0.5 | 25% | 0.19 |
| 0,66± 0.43 | 1.0± 0.7 | 0.34 | 0.5 | 0.25 | 0.165 |

In red the observed data; the numbers of the first row represent the means of the two studies leading to IFNB1a and 1b approval and used in writing the MAIN Trial protocol; in the last row the value observed in the IFNB arm by Coles et al., 2008; in black the estimated values.

The table suggests that in MAIN Trial, given the above-mentioned assumptions, it is possible to define the non-inferiority margin μ corresponding to the 25% of the mean number of observed relapses; this value is clinically relevant representing also 50% of the theoretic effect of an IFNB arm vs placebo, and it is proportionally equivalent to what established in the original protocol.

We may assume:

- 0.7±0.56 relapses /2yrs/patient, μ= 0.19; with α =0.05 and N= 69x2, β = 0.35, power = 65% (see power computation in note 1; in note 2 the power computation as in the original protocol is reported).

However, given the preliminary descriptive analysis showing in 0.7 mean overall relapses in the two arms in the 1rst year (see Addendum II), one may hypothesize the following possible setting:

- relapses/2yrs/patient = 1.4± 1.1, μ= 0.35, with α= 0.05 and N= 69x2, β = 0.40, power= 60%

Finally, if Aza efficacy was even slightly higher than IFNB, as observed in a similar study recently published (Etemadifar et al, J Neurol 2007) – so that a delta of only 0.1 could be added to μ - the following power would be obtained:

- relapses/2yrs/patient = 1.4± 1.1, μ= 0.45, with α= 0.05 and N= 69x2, β = 0.23, Power= 77%

**Conclusions**

With these new scenarios, **even with 150 patients** the **MAIN Trial maintains a 60% of probability to prove the experimental hypothesis with the primary end-point with p= 0.05**. Furthermore, the Trial maintains 80% of probability to prove the experimental hypothesis with the secondary end-point with p= 0.05 (see computations in note3)

We also point out that even if the sample were too small to prove in a conclusive way the inferiority or non-inferiority of the experimental drug versus the *comparator*, the results should be of such a good quality to be used in meta-analysis studies.

**[1] Revision of power computation for the primary end-point of MAIN Trial, according to Coles et al., 2008.**

Given the number of patients recruited in MAIN: N= 150 – 8-9 % drop-outs (expected), according to the descriptive analysis the required sample size is = 138 = 69x2**.**

Calculation of **μ** (non-inferiority margin)

Mean N of relapses/2yrs/patient ± SD observed in the IFNB = 0.72 ± 0.56

Mean N of relapses/ 2yrs/patient ± SD estimated in the placebo arm = 1.1 ± 0.86

Estimated Effect $_{\text{IFNB - placebo}}$ : – 0.38 95% CI 0.16 – 0.56

According to the EMEA guidelines on equivalence/non-inferiority studies:

μ = 95% CI: = 0.19

**-Power computation given the recruited sample**

| | |
|---|---|
| **Hypothesis 0:** | **E $_{\text{IFN-Aza}}$ = 0** |
| **μ :** | **0.19** |
| **Estimated relapses SD/2yrs IFNB arm:** | **0.56** |
| **Estimated relapses SD/2yrs Aza arm:** | **0.56** |
| **N= 150 x 0.915 * = 138/2= 69** | **69x2** |

***8-9%** *probable final drop-outs estimated at first year of study*

$$N= 2\left(\frac{Z\alpha-Z\beta \times 0.56}{x}\right)^2$$

by substituting

N = **69**

Z α = **1.64** (one-tailed) α (probability to exclude a true inferiority) = 0.05

Zβ = **- X** (onte-tailed)

$$69 = 2\left(\frac{1.64 + X \,.\, 0.56}{0.19}\right)^2$$

$$X = \frac{0.19}{0.56} \times \sqrt{\frac{69}{2}} - 1.64 = 0.35$$

Zβ (one-tailed) 0.35 = β = 0.36

**Power= 64 %**

From this revision, therefore, it appears the actual feasibility of the study with a power of 60 to 65% for the primary end-point and 80% for the secondary end-point. This means that in the new expected experimental conditions even with a smaller sample size, the MAIN Trial should mantain 60-65% of probability to observe a non-inferiority with p= 0.05.

[2] **Power Computation based on MAIN Trail Protocol approved by AIFA, based on historical RCTs (IFN beta 1b in SMRR, PRISMS Study):**

**Computation of μ** (non-inferiority margin):

Mean number of relapses 2yrs/pat. ± SD IFNB arm: 1.74

Mean number of relapses 2yrs/pat. ± SD Placebo arm: 2.55

$E_{IFN\text{-}Placebo}$ = 0.8 ± 0.4 95% CI ( = 0.5 IFN)

According to the EMEA guidelines on equivalence/non-inferiority studies μ = 95% CI $E_{comparatori\text{-}Placebo}$ observed in reference studies with μ = 0.40 (equivalent to a 50% $E_{IFN\text{-}Placebo}$) is considered clinically relevant

**On this basis sample size was calculated as follows**:

| | |
|---|---|
| Hypothesis 0 : | $E_{IFN\text{-}Aza} = 0$ |
| μ | 0.4 |
| Estimated SD relapses/2yrs IFNbeta arm: | 1.4 |
| Estimated SD relapses/2yrs Aza arm: | 1.4 |

Power computation

$$N = 2\left(\frac{Z\alpha - Z\beta}{0.4} \times 1.4\right)^2$$

By substituting

$Z\alpha = 1.64$ (one-tailed) $\alpha = 0.05$

$Z\beta = -0.84$ (one-tailed) $\beta = 0.80$

$$N = 2\left(\frac{2{,}48}{0.4} \cdot 1.4\right)^2 = 139 \times 2 = 278 \quad (360 - 20\ \%\ \text{drop-out} = 278)$$

[3] **MAIN Trial power in MRI evaluation of new cerebral lesions.**

In MAIN Trial historical study of reference showed an absolute Effect of IFNbeta vs Placebo of $5.8 \pm 7.5$ (95% CI 2.88), that is 1,45% of events observed in the IFNB arm.

The ad interim evaluation of MAIN study suggests that in the case of equivalence between the two arms (i.e. AZA vs IFNB), the number of events in IFNB arm in 2 years will be 2.3, estimating the final SD = $\pm$ 3.0 (95% C.I. 1.55 - 3.01). This suggests that in a theoretic Placebo arm a number of events = $5.6 \pm 5.4$ (95% C.I. 4.0 - 7.2) would be observed with an Effect $_{\text{IFN-Placebo}}$ of 3,3 (95% C.I. 2.7 -4.9). Thus, using the C.I. value of the estimated effect as $\mu$ value we obtain $\mu = 1.6$

-Power computation given the recruited sample.

| | |
|---|---|
| Hypothesis 0: | $E_{\text{IFN-Aza}} = 0$ |
| $\mu$ : | 1.6 |
| Relapses SD /2yrs IFNBeta arm: | 3.0 |
| Relapses SD/2yrs Aza arm: | 3.0 |
| N= 95 -10%* = 86/2= 43 | 43x2 |

**probable final drop out estimated at first year of study*

$$N = 2\left(\frac{Z\alpha - Z\beta}{x} \times SD\right)^2$$

By substituting

N = **43**

$Z\alpha$ = **1.64** (one-tailed) $\alpha$ (probability to exclude a true inferiority) = 0.05

$Z\beta$ = **- X** (one-tailed)

$$43 = 2\left(\frac{1.64 + X}{1.6} \cdot 3.0\right)^2$$

$$X = \frac{1.6}{3.0} \times \sqrt{\frac{43}{2}} - 1.64 = 0.83$$

$Z\beta$ (one-tailed) 0.83 = $\beta = 0.20$

**Power= 80 %**
