# Supplementary material for: Azathioprine versus Beta Interferons for Relapsing-Remitting Multiple Sclerosis: A Multicentre Randomized Non-Inferiority Trial
Source: PLoS One. 2014 Nov 17;9(11):e113371. doi: 10.1371/journal.pone.0113371 (PMC4234663; doi:10.1371/journal.pone.0113371)
Supplement: File S1 — Methods S1, Outcomes. Brain MRI: Scan acquisition specifications. Table S1, Serious Adverse Events (SAEs). Table S2, AEs – subtypes. (DOCX) [file pone.0113371.s004.docx]

**METHODS S1**

**Outcomes**

*Brain MRI*: *Scan acquisition specifications.*

The scans were done according to the following specifications: 1) magnet power >1 Tesla; 2) acquisition specification: - slice thickness 4 mm, no inter-slice gap; - orientation of the section: axial, centred on the lower side of the intercommissural plane of corpus callosum; - matrix 256 x 256; - FOV 25.6 cm; - two excitations; 3) direct T2w sequences: - conventional Spin Echo (SE), TR 2500 ms / TE 30 ms; - Proton Density (PD), TE 100 ms; - fFLAIR, TE 120 ms/ TR 8000 ms; - Inversion Recovery (IR), 2500/ Turbo Factor 18); 5) acquisition for BBB damage evaluation : - T1w sequences (TR 500 ms/ TE 15 ms), acquired at least eight minutes after I.V. administration of contrast medium (gadolinium, Gd, 0.1 mmol/kg); 6) acquisition schedule:a) survey T1w; b) PD and T2w scan; c) contrast medium administration; d) fFLAIR scan; e) T1 spin echo scan.

Repositioning: bicommessural axial section, that had to fall on the central slice, was used as a reference. One dummy scan of each centre had to be approved by the Image Analysis Centre before starting enrolment.

**TABLES S1 and S2**

**Table S1. Serious Adverse Events (SAEs).**

| **SAE** | **n** | **Correlation with treatment**  **(any consequent discontinued intervention)** |
| --- | --- | --- |
| **AZA** |  |  |
| Abnormal blood chemistry tests^1^ | 1 | Likely correlated  (discontinued intervention for SAE) |
| Fever and leukopenia | 1 | Likely correlated  (discontinued intervention for SAE) |
| Haemoglobin reduction | 1 | Likely correlated  (discontinued intervention for other reasons) |
| Work accident | 1 | Non-correlated  (none) |
| Surgery for gallstones | 1 | Non-correlated  (none) |
| Diverticulum perforation | 1 | Non-correlated  (none) |
| **IFN** |  |  |
| Asthma | 1 | Non-correlated  (none) |
| Uterine fibroma | 1 | Non-correlated  (none) |
| Right renal colic | 1 | Non-correlated  (none) |

Abbreviations: AZA, azathioprine; IFN, interferon; PY, person-years.

^1^Leukopenia, lymphocytopenia, red blood cell reduction, haemoglobin reduction, thrombocytopenia and other abnormal blood count.

**Table S2. AEs - subtypes.^1^**

| **Event** | **AZA** (N_patients_=69, N_events_=308, PY=108) | **IFN** (N_patients_=77, N_events_=241, PY=136) | **p-value** |
| --- | --- | --- | --- |
| **Abnormal blood count - subtypes** |  |  |  |
| Leukopenia |  |  |  |
| Patients – No./PY and rate (95%CI) | 24/108 0.22 (0.14-0.33) | 11/136 0.08 (0.04-0.14) | p<0.01 |
| AEs - No./PY and rate (95%CI) | 31/108 0.29 (0.20-0.41) | 11/136 0.08 (0.04-0.14) | p<0.01 |
| Lymphocytopenia |  |  |  |
| Patients – No./PY and rate (95%CI) | 19/108 0.18 (0.11-0.27) | 10/136 0.07 (0.04-0.14) | p=0.02 |
| AEs - No./PY and rate (95%CI) | 23/108 0.21 (0.13-0.32) | 10/136 0.07 (0.04-0.14) | p<0.01 |
| Red blood cell reduction |  |  |  |
| Patients – No./PY and rate (95%CI) | 17/108 0.16 (0.09-0.25) | 3/136 0.02 (0.00-0.06) | p<0.01 |
| AEs - No./PY and rate (95%CI) | 18/108 0.17 (0.10-0.26) | 3/136 0.02 (0.00-0.06) | p<0.01 |
| Haemoglobin reduction |  |  |  |
| Patients – No./PY and rate (95%CI) | 12/108 0.11 (0.06-0.19) | 2/136 0.01 (0.00-0.05) | p<0.01 |
| AEs - No./PY and rate (95%CI) | 13/108 0.12 (0.06-0.21) | 2/136 0.01 (0.00-0.05) | p<0.01 |
| Macrocytosis |  |  |  |
| Patients – No./PY and rate (95%CI) | 13/108 0.12 (0.06-0.21) | 1/136 0.01 (0.00-0.04) | p<0.01 |
| AEs - No./PY and rate (95%CI) | 15/108 0.14 (0.08-0.23) | 2/136 0.01 (0.00-0.05) | p<0.01 |
| Thrombocytopenia |  |  |  |
| Patients – No./PY and rate (95%CI) | 2/108 0.02 (0.00-0.07) | 0/136 | - |
| AEs - No./PY and rate (95%CI) | 2/108 0.02 (0.00-0.07) | 0/136 | - |
| Other/non-specified abnormal blood count |  |  |  |
| Patients – No./PY and rate (95%CI) | 25/108 0.23 (0.15-0.34) | 15/136 0.11 (0.06-0.18) | p=0.02 |
| AEs - No./PY and rate (95%CI) | 34/108 0.31 (0.22-0.44) | 16/136 0.12 (0.07-0.19) | p<0.01 |
| **Other abnormal blood tests - subtypes** |  |  |  |
| Increase in ALT level |  |  |  |
| Patients – No./PY and rate (95%CI) | 13/108 0.12 (0.06-0.21) | 22/136 0.16 (0.10-0.24) | p=0.40 |
| AEs - No./PY and rate (95%CI) | 13/108 0.12 (0.06-0.21) | 23/136 0.17 (0.11-0.25) | p=0.33 |
| Increase in AST level |  |  |  |
| Patients – No./PY and rate (95%CI) | 10/108 0.09 (0.04-0.17) | 13/136 0.10 (0.05-0.16) | p=0.94 |
| AEs - No./PY and rate (95%CI) | 12/108 0.11 (0.06-0.19) | 14/136 0.10 (0.06-0.17) | p=0.84 |

**Table S2. AEs - subtypes.^1^ (continued)**

| **Event** | **AZA** (N_patients_=69, N_events_=308, PY=108) | **IFN** (N_patients_=77, N_events_=241, PY=136) | **p-value** |
| --- | --- | --- | --- |
| Increase in GGT level |  |  |  |
| Patients – No./PY and rate (95%CI) | 11/108 0.10 (0.05-0.18) | 6/136 0.04 (0.02-0.10) | p=0.09 |
| AEs - No./PY and rate (95%CI) | 12/108 0.11 (0.06-0.19) | 6/136 0.04 (0.02-0.10) | p=0.06 |
| Alterations in thyroid function |  |  |  |
| Patients – No./PY and rate (95%CI) | 0/108 | 6/136 0.04 (0.02-0.10) | - |
| AEs - No./PY and rate (95%CI) | 0/108 | 6/136 0.04 (0.02-0.10) | - |
| Increase in anti-thyroid antibodies |  |  |  |
| Patients – No./PY and rate (95%CI) | 0/108 | 5/136 0.04 (0.01-0.09) | - |
| AEs - No./PY and rate (95%CI) | 0/108 | 5/136 0.04 (0.01-0.09) | - |
| Increase in bilirubin level |  |  |  |
| Patients – No./PY and rate (95%CI) | 5/108 0.05 (0.02-0.11) | 2/136 0.01 (0.00-0.05) | p=0.15 |
| AEs - No./PY and rate (95%CI) | 8/108 0.07 (0.03-0.15) | 2/136 0.01 (0.00-0.05) | p=0.02 |
| Other/non-specified abnormal blood chemistry tests |  |  |  |
| Patients – No./PY and rate (95%CI) | 12/108 0.11 (0.06-0.19) | 11/136 0.08 (0.04-0.14) | p=0.44 |
| AEs - No./PY and rate (95%CI) | 14/108 0.13 (0.07-0.22) | 11/136 0.08 (0.04-0.14) | p=0.24 |
| **Other AE - subtypes** |  |  |  |
| Headache |  |  |  |
| No. of patients/PY | 7/108 | 10/136 | - |
| Depression |  |  |  |
| No. of patients/PY | 3/108 | 3/136 | - |
| Gastralgia |  |  |  |
| No. of patients/PY | 7/108 | 1/136 | - |
| Pancreatitis |  |  |  |
| No. of patients/PY | 1/108 | 0/136 | - |
| Right renal colic |  |  |  |
| No. of patients/PY | 0 | 1/136 | - |
| Work accident |  |  |  |
| No. of patients/PY | 1/108 | 0/136 | - |
| Other non-specified |  |  |  |
| No. of patients/PY | 32/108 | 32/136 | - |

Abbreviations: AZA, azathioprine; IFN, interferon; PY, person-years.

^1^All 95% CI were estimated using the exact method; p-values for AZA vs. IFN comparison were obtained through χ^2^ test with one degree of freedom for rate comparison.
